# Thermally Stable Perovskite Solar Cells by All-Vacuum Deposition

**DOI:** 10.1021/acsami.2c14658

**Published:** 2022-12-23

**Authors:** Qimu Yuan, Kilian B. Lohmann, Robert D. J. Oliver, Alexandra J. Ramadan, Siyu Yan, James M. Ball, M. Greyson Christoforo, Nakita K. Noel, Henry J. Snaith, Laura M. Herz, Michael B. Johnston

**Affiliations:** † Department of Physics, University of Oxford, Clarendon Laboratory, Parks Road, OxfordOX1 3PU, United Kingdom; ‡ Institute for Advanced Study, Technical University of Munich, Lichtenbergstrasse 2a, GarchingD-85748, Germany

**Keywords:** metal halide perovskite, vapor deposition, hole transport layer, solar cell, thermal stability

## Abstract

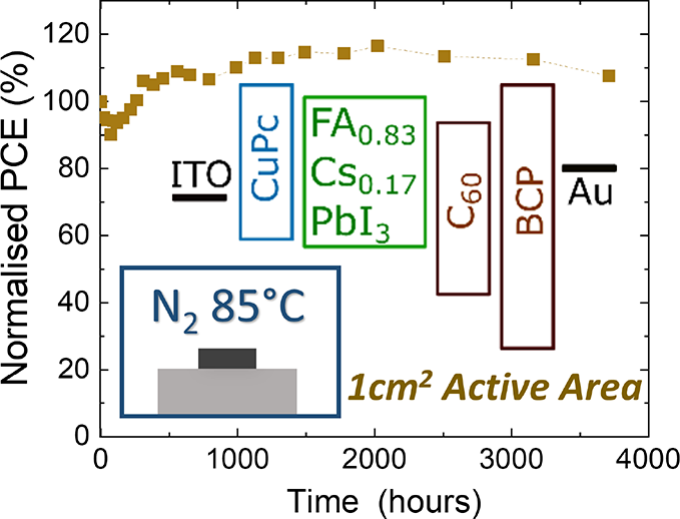

Vacuum deposition is a solvent-free method suitable for growing thin films of metal halide perovskite (MHP) semiconductors. However, most reports of high-efficiency solar cells based on such vacuum-deposited MHP films incorporate solution-processed hole transport layers (HTLs), thereby complicating prospects of industrial upscaling and potentially affecting the overall device stability. In this work, we investigate organometallic copper phthalocyanine (CuPc) and zinc phthalocyanine (ZnPc) as alternative, low-cost, and durable HTLs in all-vacuum-deposited solvent-free formamidinium-cesium lead triodide [CH(NH_2_)_2_]_0.83_Cs_0.17_PbI_3_ (FACsPbI_3_) perovskite solar cells. We elucidate that the CuPc HTL, when employed in an “inverted” p–i–n solar cell configuration, attains a solar-to-electrical power conversion efficiency of up to 13.9%. Importantly, unencapsulated devices as large as 1 cm^2^ exhibited excellent long-term stability, demonstrating no observable degradation in efficiency after more than 5000 h in storage and 3700 h under 85 °C thermal stressing in N_2_ atmosphere.

## Introduction

Since the pioneering work by Kojima et al. in 2009, MHP semiconductors have demonstrated promising potential as emerging PV materials.^1^ Because of their excellent optoelectronic properties, such as a strong absorption coefficient and a long charge carrier diffusion length,^2,3^ the certified power conversion efficiency (PCE) of single-junction MHP solar cells has currently achieved 25.7%.^4^ Additionally, with a tunable direct bandgap, MHP semiconductors are well suited for multijunction devices, including perovskite-on-perovskite and perovskite-on-silicon tandem solar cells.^5−7^

Among the wide range of techniques by which MHP thin films can be deposited, solution-processing has been the most accessible and predominant method enabling the realization of state-of-the-art laboratory-scale perovskite solar cells (PSCs).^8,9^ However, solution-processing techniques may be less pertinent for the translation to large-area industrial-scale fabrication due to costs and environmental concerns associated with solvent procurement and disposal.^10,11^ Alternatively, vacuum deposition is a solvent-free (“dry”) method whereby a source material, in the solid state, is sublimed under high vacuum, before condensing on a substrate to form a thin film of the desired material.^12^ Vacuum deposition is also a well-established technique widely used in industrial semiconductor thin-film fabrication, such as for organic light-emitting diodes and inorganic displays, which illustrates encouraging prospects for the commercial up-scaling of MHP-based devices.^10−12^ For the deposition of MHP thin films and transport layers, vacuum-based methods offer unique advantages such as precise control of layer thickness, excellent uniformity and homogeneity of the formed thin film, choice over a wide range of materials and compositions, and the flexibility to grow multilayer structures and larger-scale modules without the need to rely on complex choices of “orthogonal solvents”.^10,13−16^

PSCs with, at minimum, a thermally evaporated MHP layer have recently achieved impressive progress in their photovoltaic performance.^15,17−19^ However, the use of solution-processed HTLs is still favored in these devices, with 2,2′,7,7′-tetrakis[*N*,*N*-di(4-methoxyphenyl)amino]-9,9′-spirobifluorene (Spiro-OMeTAD), poly[bis(4-phenyl)(2,4,6-trimethylphenyl)amine] (PTAA), and [2-(3,6-dimethoxy-9*H*-carbazol-9-yl)ethyl]phosphonic acid (MeO-2PACz) as the most representative choices.^7,19−22^ For example, solution-processed Spiro-OMeTAD was employed by Feng et al. in a 21.3%-efficient PSC incorporating a layer-by-layer evaporated FA_0.85_Cs_0.15_PbI_3_ perovskite.^23^ Meanwhile, Li et al. also chose the solution-processed Spiro-OMeTAD as the HTL in their recent sequentially evaporated FA_0.95_Cs_0.05_Pb(I_1–*x*_Cl_*x*_)_3_-based PSCs, which exhibited a PCE exceeding 24%.^24^ This is in contrast to the electron transport layer (ETL), where vacuum-deposited fullerene C_60_ is one of the most commonly used materials.^20,22,25,26^ Not only are these aforementioned HTLs relatively expensive,^27^ but the additional solution-processing step also adds complication to the overall fabrication process, and thus could impede commercialization and large-scale production. Furthermore, materials such as Spiro-OMeTAD and PTAA are prone to degradation under environmental stressors such as temperature and humidity, thus curtailing the operational lifetimes of PSCs when incorporated into the device structure.^28−30^ Moreover, while the evaporated hole transporting MoO_3_/*N*4,*N*4,*N*4″,*N*4″-tetra([1,1′-biphenyl]-4-yl)-[1,1′:4′,1″-terphenyl]-4,4″-diamine (TaTm) structure has been successfully utilized in multiple high-efficiency devices by Bolink and co-workers, it is hindered by thermal instability associated with the MoO_3_ layer.^25,31^ Hence, it is paramount to find alternative HTLs that can be readily evaporated, are inexpensive, and, most critically, are stable under strict storage and operational conditions.^32^

Meanwhile, although a variety of all-vacuum-deposited PSC stacks with different ETLs and HTLs have been reported, methylammonium lead triiodide CH_3_NH_3_PbI_3_ (MAPbI_3_), formed by the vacuum codeposition of methylammonium iodide (MAI) and lead iodide (PbI_2_), has so far been the most commonly chosen photoactive semiconducting layer.^33−36^ For example, Abzieher et al. recently presented an all-evaporated MAPbI_3_ solar cell, which gave an impressive PCE as high as 19.5% by utilizing a p–i–n device architecture of ITO/2,2″,7,7″-tetra(*N*,*N*-di-*p*-tolyl)amino-9,9-spirobifluorene (Spiro-TTB)/MAPbI_3_/C_60_/Bathocuproine (BCP)/Au.^35^ However, the properties of MAI, such as its volatility, low molecular mass, and low sticking coefficient to many underlying materials of interest, make it very difficult to precisely control its deposition rate, potentially limiting the viability of vacuum-deposited MAPbI_3_.^10,37,38^ To date, the adhesion characteristics of MAI vapor have proven to be dependent on a diverse array of factors, such as deposition-chamber pressure, precursor purity, composition, and the temperature of the underlying substrates, hence complicating the coevaporation process.^20,35,38−40^ Furthermore, MAPbI_3_ degrades rapidly under thermal stress, where, for example, decomposition and emission of NH_3_ and CH_3_I gas were observed at temperatures as low as 80 °C.^41^ However, replacing the MA^+^ cation with CH(NH_2_)_2_^+^ (FA^+^) significantly improves resistance to thermal decomposition.^7,25^ Unfortunately, neat FAPbI_3_ perovskites are also susceptible to structural phase transitions.^25^ In particular, the radius of the FA^+^ cation is significantly larger than that of the MA^+^, which leads to FAPbI_3_ being only metastable in its black perovskite α-phase, with its yellow hexagonal δ-phase being thermodynamically favored at room temperature.^42^ However, alloying FA^+^ with Cs^+^ (which has a smaller cation radius) forms the stable FA_1–*x*_Cs_*x*_PbI_3_ in its perovskite α-phase for a range of alloy fractions from *x* = 0.1 to *x* = 0.5.^43,44^ Nevertheless, there are only a few reports of vacuum codeposition of such multication FACs-based perovskites.^19,23,24,26,45^

CuPc and ZnPc are organometallic semiconductors from the class of metal phthalocyanine and can be deposited through thermal evaporation.^33,46^ They are desirable HTL candidates for an all-vacuum-processed PSC due to their acceptable hole mobility (order of 10^–3^ cm^2^ V^–1^ s^–1^ for thermal-evaporated CuPc and ZnPc), well-aligned energy levels, relatively low cost, and excellent chemical and thermal stability.^46−50^ Even when CuPc derivatives are solution-processed, devices exhibit enhanced long-term stability under thermal stressing and cycling, especially when compared to PTAA or Spiro-OMeTAD.^51,52^ While evaporated PSCs with ZnPc HTL are rarely investigated,^46,53^ CuPc has been successfully employed with all-evaporated MAPbI_3_ stacks in both n–i–p and p–i–n configurations.^33,36,54^ However, the difference between CuPc and ZnPc is not yet clear, and neither has been used with vacuum-deposited FACs-based perovskite. More importantly, the long-term stability and degradation pathways are so far not well-understood for an all-evaporated stack.

Herein, we elucidate the different optoelectronic properties and device performance between CuPc and ZnPc as the HTL, when employed with coevaporated FA_0.83_Cs_0.17_PbI_3_ (bandgap of approximately 1.56 eV) in the p–i–n configuration. We also uncover the varying adhesion behavior of the organic FAI precursor on underlying substrates, which influences the stoichiometry of the deposited FACs perovskite films. We further optimized the perovskite composition on CuPc by introducing a 10% increase in PbI_2_ evaporation rate from the stoichiometric rate of 0.3 Å/s and improved the all-vacuum-deposited device up to a PCE of 13.9%. Finally, we thoroughly examined their long-term stability under a range of testing conditions and demonstrated that devices are intrinsically stable for more than 3700 h (154 days) under 85 °C thermal stressing.

## Results and Discussion

### Comparison of Hole Transport Layers

**Figure 1:**
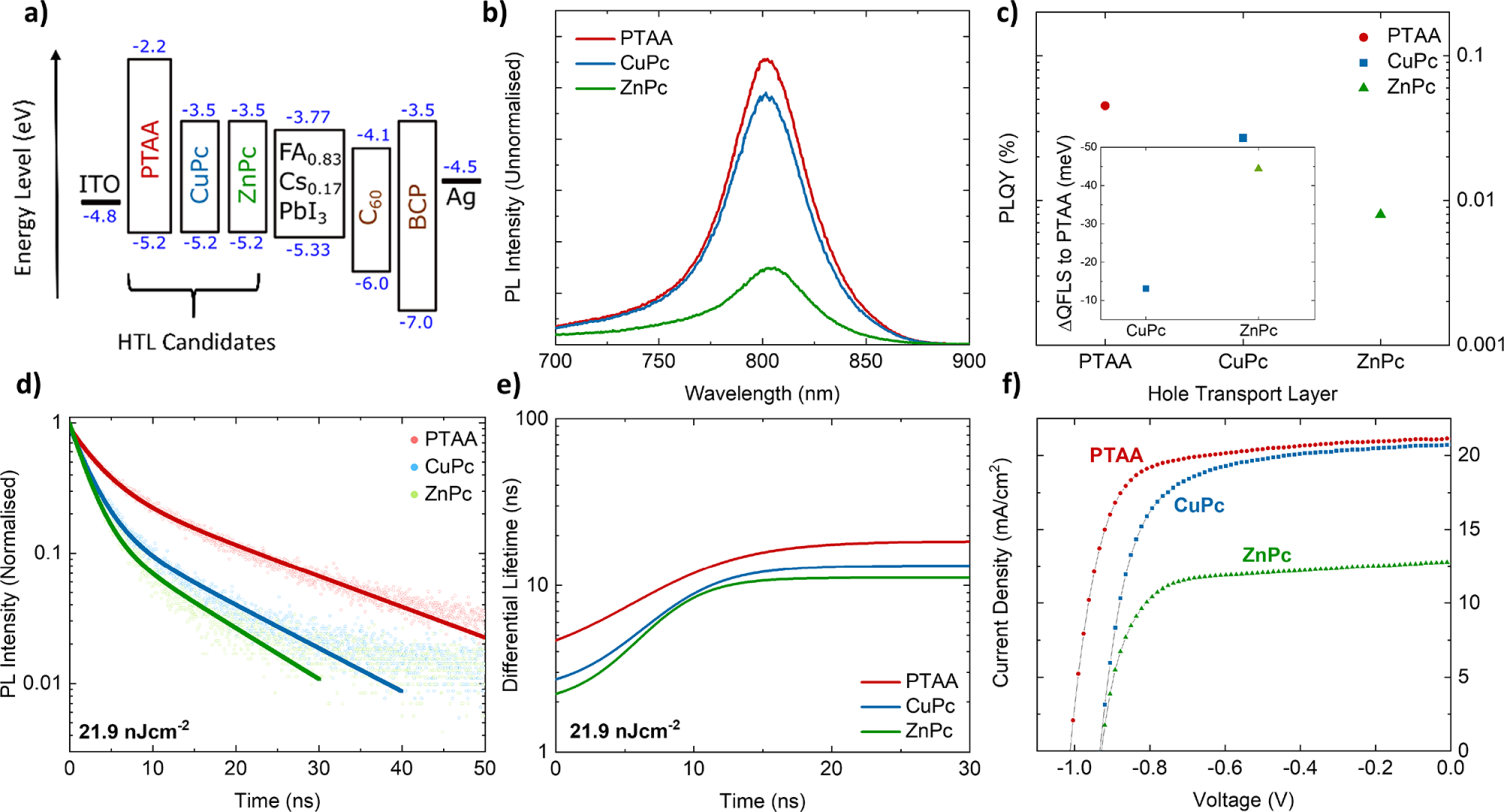
A comparison of the optoelectronic properties and device performance for the different hole transport layers (HTLs) of PTAA, CuPc, and ZnPc. (a) Schematic diagram of energy levels from literature reports of the investigated device stack.^33,34,36,46,55,56^ (b) Unnormalized photoluminescence (PL) spectra of the ITO/HTL/FA_0.83_Cs_0.17_PbI_3_ half stack from photoexcitation of a 398 nm laser. (c) Photoluminescence quantum yield measurement of the same half stack; the inset illustrates the expected quasi-Fermi level splitting difference between PTAA and CuPc or ZnPc. (d) Time-correlated single photon counting measurement of the same half stack by excitation with a 398 nm pulsed diode laser at a repetition frequency of 10 MHz, fitted with a double exponential decay (solid lines). (e) Calculated differential lifetime from transient PL decay using eq 2. (f) Reverse scan from current–voltage measurements of the best ITO/HTL/FA_0.83_Cs_0.17_PbI_3_/C_60_/BCP/Ag devices.

We investigated both evaporated CuPc and ZnPc as potential HTL candidates in an all-evaporated p–i–n stack with the following configurations of ITO/HTL (CuPc or ZnPc)/FA_0.83_Cs_0.17_PbI_3_/C_60_/BCP/metal electrodes (Ag or Au). Reference devices were fabricated using solution-processed PTAA as HTL. Figure 1a illustrates the band-energy diagram of the investigated device stack according to literature values.^33,34,36,46,55,56^ Both CuPc and ZnPc are direct-gap semiconductors with a bandgap of approximately 1.7 eV.^33,36,46^ Similar to PTAA, previous studies have suggested CuPc has a highest occupied molecular orbital (HOMO) energy of −5.2 eV,^33,47^ which is well-aligned with that of the evaporated FA_0.83_Cs_0.17_PbI_3_ perovskite and would facilitate efficient hole extraction with minimal loss in the open-circuit voltage (*V*_oc_). Indeed, transmission-reflection measurements of CuPc and ZnPc thin films of various thickness deposited on z-cut quartz substrates indicate similar transmission and absorption characteristics (Figure S1). Thicker 300 nm films of both CuPc and ZnPc absorb strongly in the green to red region (Figure S1e,f), which is expected from their comparable bandgaps and is consistent with previous reports.^46,48^

We first examined solar-cell “half stacks”, which consisted of ITO/HTL/FA_0.83_Cs_0.17_PbI_3_. Figure 1b outlines their respective photoluminescence (PL) spectra and shows no significant shift in the emission wavelength, indicating that a perovskite with similar stoichiometry has formed on all HTLs. Moreover, the measured PL is much more intense in samples with PTAA as HTL than with ZnPc. Although often a reduction in PL intensity can be correlated with the quenching effect,^57^ we believe that this observation is consistent with a higher nonradiative recombination center density at the ZnPc–perovskite interface as compared to the PTAA–perovskite control. This notion is further supported by findings from photoluminescence quantum yield (PLQY) measurements, which quantify the degree of nonradiative recombination. Figure 1c shows that, while the PLQY values are comparable between PTAA (0.045%) and CuPc (0.037%), the PLQY of ZnPc (0.008%) is particularly poor and on the boundary of the instrument response. This suggests significant nonradiative recombination at the ZnPc–perovskite interface, evincing that the initial perovskite interface may have grown poorly on ZnPc. The inset of Figure 1c shows an interfacial comparison of the change in quasi-Fermi level splitting (QFLS) between PTAA and metal phthalocyanine (Pc) HTLs:
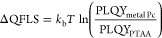
1where *k*_b_ is the Boltzmann constant and *T* is approximated to room temperature.^58,5900^ As the perovskite bandgap is unchanged on each HTL, this bandgap-independent analysis deduces that the expected decrease in QFLS, or equivalently *V*_oc_, is much more significant between PTAA and ZnPc than that between PTAA and CuPc, by more than 30 meV.

Furthermore, a similar optoelectronic response is identified from time-resolved PL measurements of these half stacks using the time-correlated single photon counting (TCSPC) technique (Figure 1d), with additional TCSPC transients from different excitation fluences plotted in Figure S4. We analyzed the transient PL decay with the method previously reported by Oliver et al.^58^ A double exponential decay was used for reproducing the transients and accounting for different electron–hole recombination processes.^59,60^ We then calculated the differential lifetime by taking the numerical derivative of the fitted photon flux (Φ(*t*)) according to
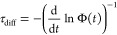
2

As shown in Figure 1e, early rises in the differential lifetimes most likely correspond to the charge transfer from the HTL upon photoexcitation. At larger delay time, further electron–hole recombination is dominated at the interface of HTL–perovskite. The use of PTAA as HTL corresponds to the longest differential lifetime at a large delay time, followed by CuPc. This indicates reduced nonradiative recombination at the interface of CuPc–perovskite as compared to that of ZnPc–perovskite, in agreement with our steady-state PL and PLQY measurements. As such, we would expect CuPc to work better than ZnPc in a solar cell as HTL.

We fabricated complete p–i–n solar-cell devices to examine the compatibility of these three HTLs with our vacuum codeposited FA_0.83_Cs_0.17_PbI_3_ perovskite. The current–voltage (*J*–*V*) curves from reverse scans of the best devices are plotted in Figure 1f, and the device performance statistics are shown in Figure S5. These results illustrate that solar cells with solution-processed PTAA HTL performed best with PCE as high as 15.3%, mainly as a result of a higher *V*_oc_, which may be attributed to its lowest unoccupied molecular orbital being higher than those of CuPc and ZnPc (see Figure 1a) and thus more favorable to electron-blocking. However, there is more pronounced hysteresis observed in devices with PTAA as HTL than with CuPc (Figure S6). From comparison between devices with CuPc and ZnPc, we find a small mean difference of 0.05 V in *V*_oc_, as expected from our optical measurements. Interestingly, there is a significant improvement in short-circuit current density (*J*_sc_) of almost 5 mA/cm^2^ for CuPc devices as compared to ZnPc devices. This implies that the difference between CuPc and ZnPc is more nuanced than just interfacial properties and is likely to be also related to the reduced optical transmission of ZnPc (Figure S1) as well as a lack of charge selectivity. Indeed, poor external quantum efficiency (EQE) is seen for full solar cells with ZnPc HTLs, indicating increased parasitic absorption from the ZnPc HTL as well as poor electron extraction from these devices (Figure S7).

The strongly increasing optical absorption coefficient of FA_0.83_Cs_0.17_PbI_3_ as the wavelength is reduced results in the bluer light generating electron–hole pairs very close to the HTL interface in solar cells with a p–i–n architecture.^22^ This means holes are more readily extracted due to the short distance to the electrode, whereas electrons have to traverse most of the device depth. The drop in EQE at wavelength <500 nm for solar cells with ZnPc HTLs as compared against those with CuPc HTLs indicates that electrons recombine rapidly at the FA_0.83_Cs_0.17_PbI_3_–ZnPc interface or are extracted into the ZnPc and recombine there. Nevertheless, these results suggest that CuPc would be a more proficient HTL than ZnPc, and hence we focused on optimizing an all-evaporated device with CuPc as the HTL.

### Adhesion Characteristics of Evaporated Perovskite Precursors

**Figure 2:**
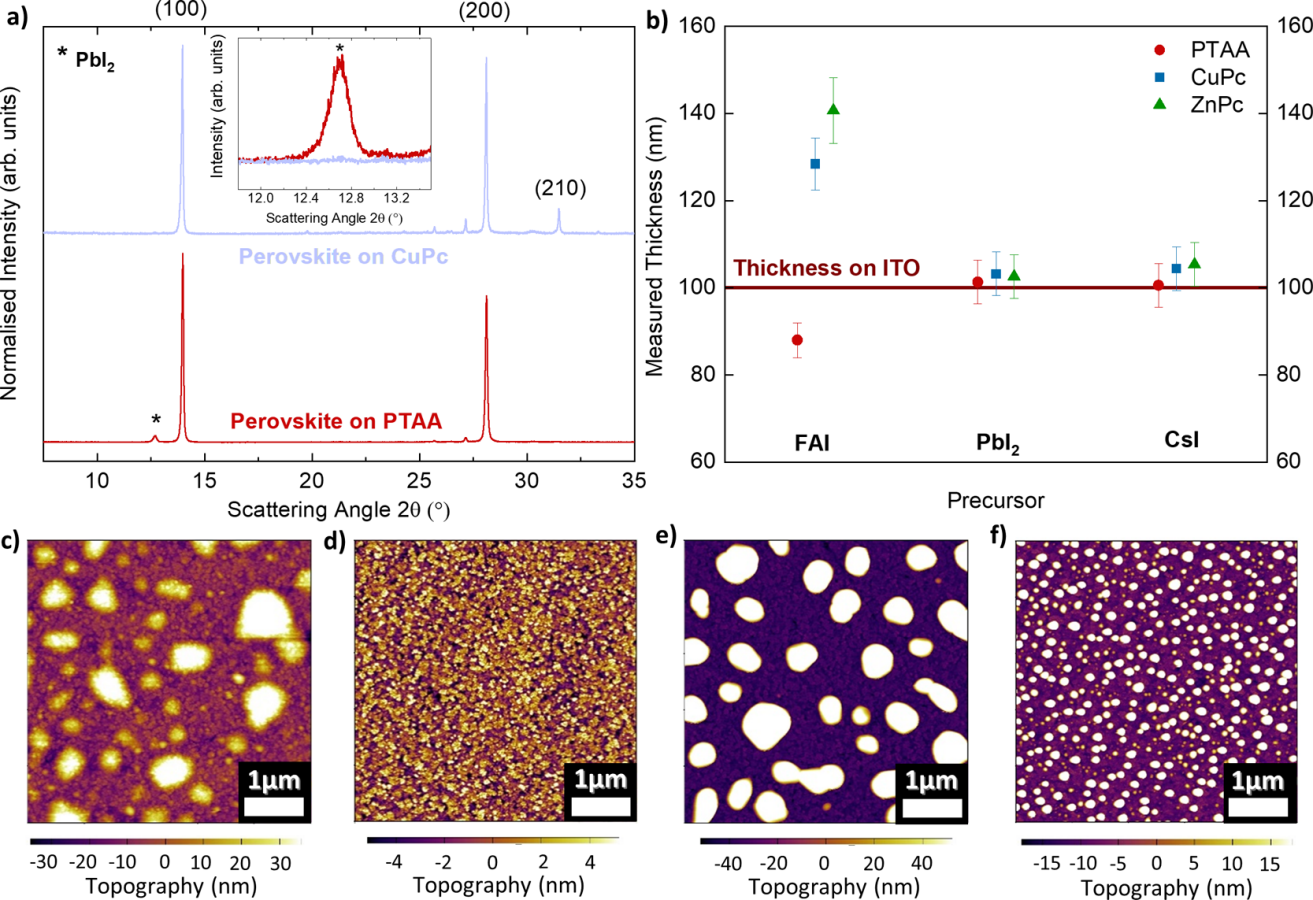
Variation in FAI adhesion characteristics to different underlying layers and impact on the perovskite crystalline phases. (a) Normalized X-ray diffraction (XRD) pattern of the ITO/HTL/FA_0.83_Cs_0.17_PbI_3_ half stack, where HTL is CuPc (blue) or PTAA (red). The inset illustrates the zoomed-in and unnormalized PbI_2_ diffraction peaks. The X-ray source was Cu K_α_ λ = 1.54 Å, and each spectrum was corrected for sample displacement. (b) Measured thickness from a stylus profiler of FAI, PbI_2_, and CsI precursors each deposited with the same time-integrated vapor flux on different HTLs and ITO substrate. Each precursor’s deposition time is controlled such that a 100 nm thick layer is formed on the ITO substrate. (c and d) Atomic force microscopy (AFM) images of a thin 5 nm FAI film deposited on CuPc and PTAA, respectively. (e and f) AFM images of a thin 25 nm FAI film deposited on CuPc and PTAA, respectively. For all AFM measurements, the thickness of the deposited FAI films was controlled by the tooling factor of FAI on PTAA.

Precise control of deposition parameters is critical in forming highly crystalline perovskite films with correct phases in vacuum codeposition and hence achieving optimal solar-cell performance. In particular, multiple past reports have demonstrated that the underlying substrate is crucial in influencing the growth of perovskite films, especially with coevaporated MAPbI_3_.^35,39,61^ Yet, there have been few investigations of the deposition mechanism of organic FAI vapor to date, which, as for MAI, may prove pivotal in controlling perovskite-film growth. Therefore, we first endeavor the understanding of the growth of FA_0.83_Cs_0.17_PbI_3_ on the CuPc HTL. We vacuum codeposited FA_0.83_Cs_0.17_PbI_3_ perovskite simultaneously on substrates of PTAA and CuPc and investigated their respective crystalline phases via X-ray diffraction (XRD). Figure 2a indicates that the perovskite deposited on PTAA exhibits subtle differences from that on CuPc, despite similar (100) and (200) perovskite diffraction peaks. In particular, the PbI_2_ peak at 2θ of 12.7° is absent for perovskite formed on CuPc. Additionally, a small peak at 32°, which corresponds to the (210) peak, is only seen from the perovskite formed on CuPc, suggesting a slightly disparate orientation of the perovskite as a result of different templating from underlying transport layers.

To probe the difference in the crystalline phases of coevaporated perovskite, we first measured the tooling factor of all precursors on different HTLs, which can convey important understandings of their adhesion characteristics.^20^ The measured results are summarized in Table S1. Here, we define the tooling factor as the ratio of the actual film thickness measured via a stylus profiler to the registered thickness from the quartz crystal microbalance near the substrate. Figure 2b illustrates the measured thickness of each precursor deposited on different HTLs with reference to a controlled 100 nm thick layer deposited on cleaned ITO substrates, respectively. Strikingly, the measured FAI thicknesses and tooling factors vary significantly across the different HTLs, with a 50% thicker FAI film being deposited on CuPc as opposed to PTAA. This observation clearly demonstrates that the sticking properties of FAI vary distinctively across different underlying surfaces. In comparison, the adhesion characteristics of the inorganic precursors, CsI and PbI_2_, are essentially unaltered on different HTLs, as reflected by the consistent tooling factors and similar thicknesses measured across different substrates. These data are shown in Figure 2b and Table S1.

To elucidate this further, we performed atomic force microscopy (AFM) on FAI thin films of 5 and 25 nm deposited on CuPc and PTAA (Figures 2c–f and S8). The different FAI thicknesses were controlled by its tooling factor on PTAA. While it is evident that FAI adopts an “island growth” mode on both PTAA and CuPc, contrasting nucleation dynamics are observed. On PTAA, FAI nucleates across the film forming a significant number of small islands, which is reflected by the smooth film with no considerable topological variation (Figure 2d and f). This is juxtaposed with the sticking mechanics of FAI on CuPc shown in Figure 2c and e, where it is seen that the FAI vapor preferentially binds to itself and forms large islands with substantial undulation. This also suggests that the increased tooling factor of FAI on CuPc results from the initial FAI nucleation on CuPc; that is, FAI sticks better to itself and hence forms a thicker layer overall on CuPc.

Interestingly, a few previous works have suggested that the adhesion of FAI vapor is independent of the chemical composition of the underlying substrates.^25,26^ Indeed, from AFM and scanning electron microscopy, the different FAI sticking dynamics may not influence the surface morphology of a coevaporated perovskite layer as thin as 25 nm (Figures S9 and S10). However, as seen in the XRD data in Figure 2a, the change in the FAI tooling factors between PTAA and CuPc has inevitably influenced the stoichiometry of the resulting 520 nm thick perovskite layer. This finding also explains the absence of a PbI_2_ peak in the XRD pattern for the perovskite layer deposited on CuPc (Figure 2a) as more FAI had adhered to the CuPc surface initially as “large islands” and reacted with PbI_2_ and CsI. Therefore, we believe that if FAI is to be used as a precursor in any coevaporated perovskite, it is important to optimize the perovskite stoichiometry specific to different transport layers.

Hereon, we refer to FA_0.83_Cs_0.17_PbI_3_ perovskite deposited on CuPc with no PbI_2_ excess as the “stoichiometric” case (Figure 2a). From the distinctive sticking behavior of FAI on CuPc, we further optimized the perovskite composition by introducing a controlled amount of excess PbI_2_ through increasing the PbI_2_ deposition rate from the stoichiometric rate of 0.3 Å/s, while maintaining all other precursors’ deposition rates (full parameters are available in the Supporting Information). Figure S12 shows that the intensity of the PbI_2_ XRD peak at 12.7° increases with increasing PbI_2_ deposition rate, indicating that unreacted PbI_2_ has been introduced into the perovskite film.

### Device Optimization with the CuPc Hole Transport Layer

**Figure 3:**
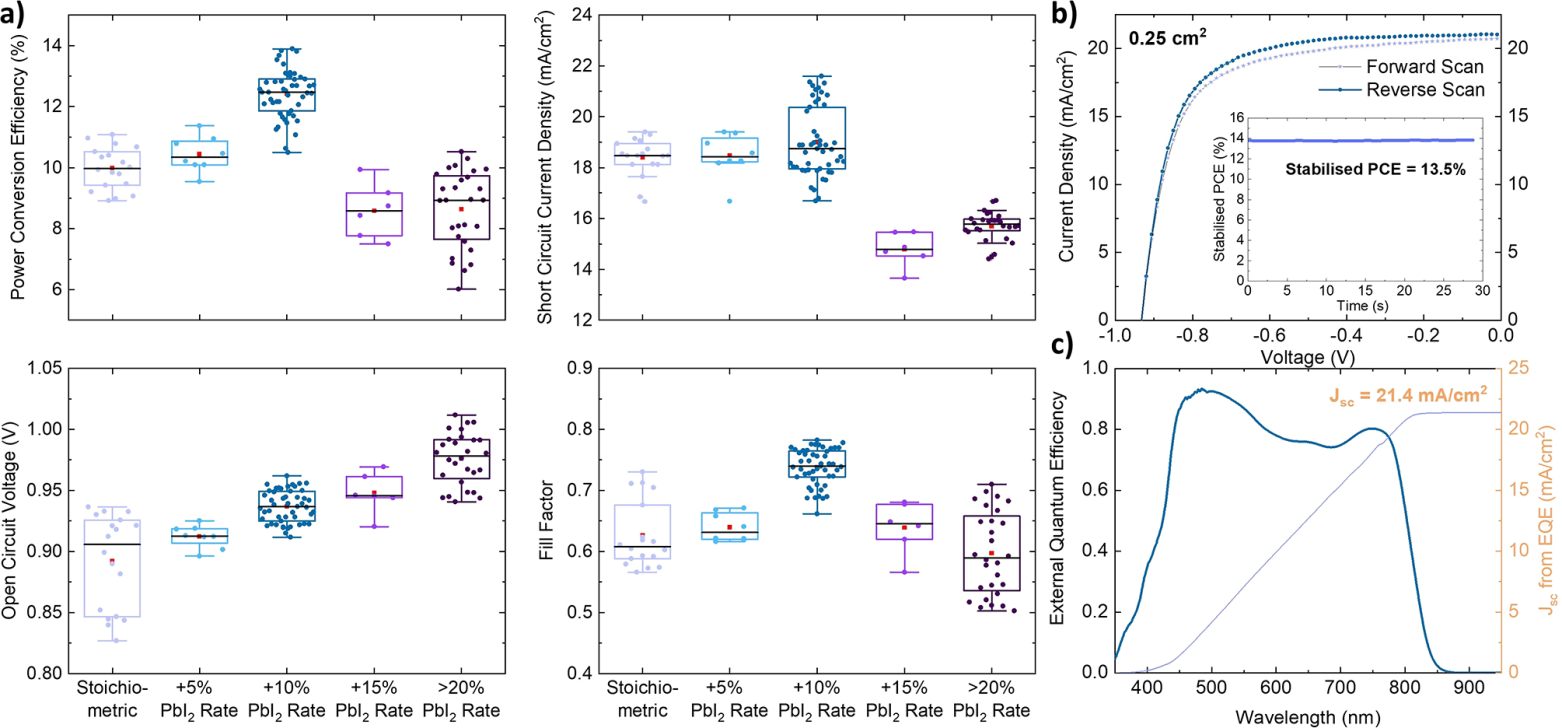
Optimization of all-evaporated ITO/CuPc/FA_0.83_Cs_0.17_PbI_3_/C_60_/BCP/Ag devices. (a) Photovoltaic performance boxplots from reverse current–voltage (*J*–*V*) scans for perovskites with different PbI_2_ evaporation rates during the coevaporation. Mean and median are denoted by an orange “■” and a black line, respectively. (b) *J*–*V* curves of the 0.25 cm^2^ active area champion device with optimized 10% excess PbI_2_ rate for the coevaporated FA_0.83_Cs_0.17_PbI_3_ with the inset showing its stabilized power output, tracked near the maximum power point for 30 s. (c) External quantum efficiency spectrum and calculated short-circuit current density (*J*_sc_) of the same champion device. The data in (a) and (b) have been corrected for spectral mismatch between the solar spectrum and the solar simulator used in these experiments, as detailed in section 1.2 of the Supporting Information.

Figure 3a illustrates the photovoltaic performance of all-vacuum-deposited solar-cell devices with the structure of ITO/CuPc (7.5 nm)/FA_0.83_Cs_0.17_PbI_3_ (520 nm)/C_60_ (23 nm)/BCP (2 nm)/Ag (100 nm). Solar cells with stoichiometric FA_0.83_Cs_0.17_PbI_3_ perovskite deposited on CuPc exhibit a large spread in device performance and have an undesirably low *V*_oc_ of around 0.9 V, which limits the PCE output to between 9% and 11%. With more PbI_2_ evaporated, a constant improvement in *V*_oc_ can be achieved, and there is an almost 100 mV boost between coevaporating perovskite with the stoichiometric parameters and when the PbI_2_ rate is increased by more than 20% excess. This trend of *V*_oc_ boost is consistent with a previous report of coevaporated FACs-based perovskites.^19^ However, the measured *J*_sc_ quickly deteriorates when the PbI_2_ rate is increased beyond 15% excess. We found that the best devices are attained with an increment of 10% excess PbI_2_ rate from the stoichiometric case, which resulted in a *V*_oc_ boost, in conjunction with a small enhancement in both *J*_sc_ and fill factor (FF).

Forward and reverse *J*–*V* scans under simulated solar conditions (1 kW/m^2^, AM1.5) of the champion all-evaporated device (0.25 cm^2^ active area) are plotted in Figure 3b. A PCE of 13.9% (from the max power point), *J*_sc_ of 21.1 mA/cm^2^, *V*_oc_ of 0.93 V, and FF of 0.72 were observed. Notably, minimal hysteresis is observed in the champion 0.25 cm^2^-area all-evaporated device with CuPc HTL. The inset of Figure 3b shows the stabilized PCE under continuous illumination for 30 s, averaging around 13.5%. The efficiency of the 1 cm^2^-area device is slightly lower than that of the 0.25 cm^2^-area device due to further loss in the FF. The best 1 cm^2^-device has a PCE of 12.9% and a stabilized output of 12.5% (Figure S13).

Figure 3c displays the EQE spectrum of the champion 0.25 cm^2^-area device and the corresponding integrated *J*_sc_. The EQE spectrum demonstrates a strong peak in the blue region, indicating electrons are well-extracted. We conclude that the dip at 600 nm wavelength is associated with the CuPc absorption (Figure S1), as expected for a solar cell in the p–i–n configuration. An integrated *J*_sc_ of 21.4 mA/cm^2^ was calculated from EQE of the photovoltaic device, in excellent agreement with the measured *J*_sc_ of 21.1 mA/cm^2^ from the *J*–*V* scan.

### Device Stability

The stability of solution-processed PSCs has been studied previously under a range of environmental and operational conditions.^62^ Remarkably, however, similar rigorous benchmarks are scarcely reported for (all-)evaporated perovskites and devices.^63^ Here, we examined a range of storage conditions for the optimized all-evaporated stack with different metal contacts of Ag or Au.

**Figure 4:**
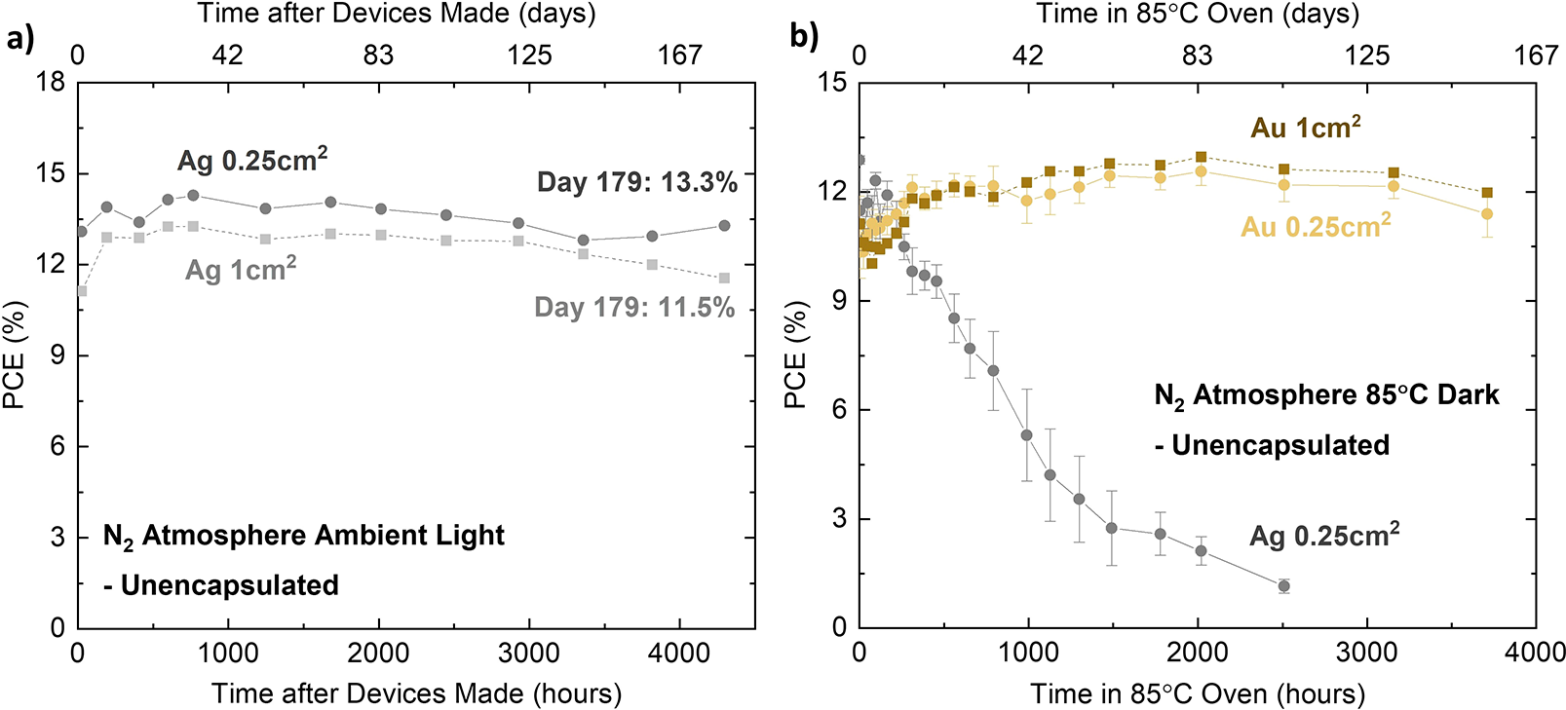
Stability studies of optimized all-evaporated ITO/CuPc/FA_0.83_Cs_0.17_PbI_3_/C_60_/BCP/metal contact devices. (a) Unencapsulated devices capped with Ag contacts were stored in N_2_ atmosphere and under ambient light. Day 1 PCE values of 13.1% (0.25 cm^2^) and 11.2% (1 cm^2^) were measured. (b) Unencapsulated devices were kept in an 85 °C oven in N_2_ atmosphere in the dark. For devices with Ag contacts, the averaged PCE values of five devices are presented (gray “●”). For 0.25 cm^2^-active area devices with Au contact, averaged PCE values of six devices are presented up to 1298 h, and those of three devices are shown afterward (yellow “●”). One Au device with a 1 cm^2^-active area, which recorded a PCE of 11.2% initially, is also plotted (brown “■”). All data points have been corrected for spectral mismatch between the solar spectrum and the solar simulator used in these experiments, as detailed in section 1.2 of the Supporting Information.

First, the shelf-life stability in N_2_ atmosphere of the 0.25 and 1 cm^2^ champion devices, fabricated in the same batch, was tracked over time (unnormalized in Figure 4a and normalized in Figure S14). Regular testing identified that the PCE of the photovoltaic devices had improved slightly over the first 720 h (30 days), before maintaining 104% and 101% of their initial efficiencies after 4296 h (179 days), respectively. A second EQE measurement was also taken on the champion 0.25 cm^2^-area devices after 3192 h (133 days), revealing only a slight change in the spectra and a similar integrated *J*_sc_ (Figure S15). We further demonstrate a consistent device stability trend by measuring additional 0.25 and 1 cm^2^-area devices made in the same and in separate batches and stored in the same environment (Figures S16 and S17). Figure S17 highlights that all devices were able to maintain their initial performance output for a period in excess of 5000 h (208 days), with the longest tested devices retaining more than 90% of the initial PCE exceeding 7200 h (300 days), demonstrating excellent batch-to-batch reproducibility. We also note that the larger 1 cm^2^-area devices exhibit shelf stability behavior similar to that of all 0.25 cm^2^-area devices, underlining the outstanding uniformity and homogeneity of this all-vacuum-deposited stack. The spread in device performance increases after 5500 h, after which a few devices ceased to operate, which is likely a result of corrosion of the Ag contacts. The stability of the PTAA devices within a N_2_ atmosphere is also studied as a reference (Figures S18 and S19). PTAA devices also demonstrated an excellent shelf-life stability, with all 0.25 cm^2^-area devices retaining more than 90% of their respective initial efficiency after 2800 h (117 days). Nevertheless, the larger 1 cm^2^-area device quickly failed only after 648 h (27 days), which may have succumbed to reactions with the presence of residual solvent.

In parallel, Figures 4b, S20a, and S21 demonstrate the impressive thermal stability of these unencapsulated devices when placed inside an 85 °C oven in N_2_ at atmospheric pressure in darkness. Devices with active areas of 0.25 and 1 cm^2^ capped with Au contact retained more than 100% of their original PCE after more than 3710 h (154 days), despite experiencing an early dip in their performance. These findings suggest that there is minimal thermal-induced degradation of any element of the device stack.

We also thermally stressed devices capped with Ag metal contacts at 85 °C in N_2_. For solution-processed PSCs, it has been reported that I^–^ ions can migrate through the ETL and react with Ag electrodes to form AgI, undermining the stability of the devices.^64,65^Figures 4b and S20b depict that the majority of Ag devices maintained a minimum 95% of initial performance for the first 200 h, before experiencing a gradual decay, and after 1 month in the oven, only recorded between 60% and 75% of their initial efficiencies. Hence, this operational stability testing accentuates the excellent thermal stability of devices with Au contacts, which we attribute to the use of CuPc HTL and MA-free FACs perovskite. In particular, the high sublimation temperature (>300 °C) and decomposition temperature (>530 °C) of CuPc have resulted in its enhanced thermal durability as a stand-alone material and in perovskite photovoltaic devices.^36,51,66^

Furthermore, unencapsulated devices were also kept in a dark ambient condition (30–40% relative humidity) to probe their response to a combination of air and moisture. After nearly 1500 h, the majority of devices with Au electrodes maintained more than 80% of their initial PCE, with the smaller active-area devices outperforming the larger area ones (Figure S22), before eventually succumbing to moisture- or oxygen-related degradation. In comparison, devices with Ag contacts failed much quicker. XRD patterns (shown in Figure S23) illustrate that device degradation was most likely caused by a phase transition into the yellow hexagonal δ-phase for the perovskite layer.

## Conclusion

We have investigated the use of metal phthalocyanines as cost-effective and stable hole-transporting layers in all-vacuum-deposited MA-free p–i–n perovskite solar cells. We studied the nucleation and growth of the vacuum-deposited FA_0.83_Cs_0.17_PbI_3_ perovskite on two vacuum-deposited metal phthalocyanines (CuPc and ZnPc) and on a control solution-processed hole transport layer (PTAA). In particular, we found striking differences in the sticking, adhesion, and nucleation of the perovskite precursor, FAI, to the metal phthalocyanines layer in comparison with the PTAA layer. This highlights the impact of sticking coefficients on the stoichiometry of vacuum codeposited perovskite films and hence the importance of optimizing growth parameters of FA-based MHPs by vacuum processes on different materials. We also demonstrate that the incorporation of a small amount of PbI_2_ excess in the coevaporation of FA_0.83_Cs_0.17_PbI_3_ can enhance device performance, in particular, with *V*_oc_ improvement.

We find that all-vacuum-deposited solar cells based on intrinsic FA_0.83_Cs_0.17_PbI_3_ semiconducting thin films with CuPc as hole-transporting layers consistently outperformed those based on ZnPc despite their similar electronic structure. We conclude that the higher rates of electron–hole recombination in the vicinity of the ZnPc–perovskite interface are the likely cause of the inferior performance of solar cells with ZnPc as the hole transport layer.

Significantly, we demonstrate an impressive thermal stability for the all-vacuum-deposited p–i–n solar cells with CuPc as the hole transport layer and FA_0.83_Cs_0.17_PbI_3_ as the intrinsic semiconductor. These solar cells exhibited excellent durability of more than 150 days under thermal stressing and long-term storage conditions, with reasonable PCEs of just below 14%. In addition, a similar stability was observed for larger 1 cm^2^-active-area devices, epitomizing that CuPc is a viable, cheap, and scalable hole transport layer. Looking forward, CuPc is a promising thermally stable component for more complicated solar-cell architectures, such as tandem and multilayer solar cells, which are well suited to fabrication by all-vacuum deposition.

## Experimental Methods

### Device Fabrication

ITO substrates were gently brushed in Decon-90 detergent solution (1% volume in deionized water) and then sonicated for 5 min each in fresh Decon-90 solution, deionized water, acetone, and isopropyl alcohol sequentially. Before deposition, the substrates were further treated with UV-Ozone for 15 min.

To fabricate solar-cell devices, the HTLs of CuPc and ZnPc, the perovskite layer, the electron transport layer C_60_, and the buffer layer BCP were all evaporated in the same custom-built thermal evaporator. The chamber was pumped down to a base pressure between 8 × 10^–7^ and 2 × 10^–6^ mbar for all depositions. The walls of the chamber were maintained at 17 °C and the rotating substrate at 20 °C through two separate chillers. Rates were monitored through gold-plated quartz crystal microbalances (QCMs) and a customized control software. During all depositions, QCM readings at each source and at the substrate were cross-checked. Each precursor material was individually calibrated on cleaned ITO substrates (or other underlying layers, where applicable) to determine the tooling factor, and hence the actual deposition rate.

CuPc (Sigma-Aldrich, >99.95% trace metal basis, triple-sublime grade) was evaporated at a rate of 0.08 Å/s at temperatures between 320 and 340 °C until a layer thickness of 7.5 nm was achieved. ZnPc (Lumtec, >99%, sublime grade) was evaporated at a rate of 0.08 Å/s at temperatures between 300 and 330 °C until a layer thickness of 7.5 nm was achieved.

The PTAA transport layer was solution-processed in a glovebox under N_2_ atmosphere. The PTAA powder (Xi’an Polymer Light Technology) was dissolved in toluene solvent at a concentration of 1.5 mg/mL, stirred overnight, and spin-coated statically with 100 μL of solution at 6000 rpm for 30 s with an acceleration of 2000 rpm, followed by 10 min annealing at 100 °C on a hot plate in the same N_2_ atmosphere.

For the perovskite layer, FAI (Dynamo, 99.999%), PbI_2_ (Alfa-Aeser, 99.998% metal base), and CsI (Alfa-Aeser, 99.998% metal base) were coevaporated to form the FA_0.83_Cs_0.17_PbI_3_ composition. To form the precise stoichiometric FA_0.83_Cs_0.17_PbI_3_ composition on CuPc, FAI was evaporated at 0.2 Å/s (155–170 °C), PbI_2_ was evaporated at 0.3 Å/s (270–290 °C), and CsI was evaporated at 0.04 Å/s (400–430 °C). For each deposition, FAI powder in the crucible was topped up to 1.1 g, and for a 520 nm perovskite layer, 0.19–0.21 g of FAI was typically evaporated. All as-deposited films were further annealed at 135 °C for 30 min on a hot plate under N_2_ glovebox conditions.

Fullerene C_60_ (Acros Organics, 99.9%) was deposited at 0.1 Å/s to form a 23 nm thick layer for devices with Ag contact, and a 30 nm thick layer for devices with Au contact. Subsequently, a 2 or 5 nm thick BCP (Sigma-Aldrich, 99.5%) layer was deposited at 0.07 Å/s for devices with Ag contact or Au contact, respectively.

The Ag top contact with a thickness of 100 nm was evaporated in a separate Lesker Nano36 chamber. Using QCM readings, we maintained the initial rate at 0.2 Å/s for the first 10 nm, before ramping up to 1.5 Å/s. The Au top contact with a thickness of 100 nm was evaporated in the same Lesker Nano36 chamber. The initial rate was maintained at 0.1 Å/s for the first 10 nm, before ramping up to 0.7 Å/s.

### Solar Cell Characterization

Devices were measured under stimulated AM1.5G sunlight with an equivalent irradiance of 100 mW/cm^2^, generated by a Wavelabs Sinus-220 solar simulator and a Keithley 2400 source meter. The solar simulator was calibrated with respect to a KG-3 filtered silicon reference photodiode (Fraunhofer) prior to the measurement. Devices were characterized in ambient air conditions at room temperature with relative humidity between 25% and 40%. The *V*_oc_ was first measured for 3 s. Reverse and forward scans between −1.2 and 0.2 V at a constant scan rate of 0.13 V/s were sequentially performed. Steady-state current and voltage were further probed for 30 s under continuous illumination, keeping the device close to its maximum power point (MPP) by actively tracking the maximum power point with a gradient descent algorithm. Finally, *J*_sc_ was measured for 3 s. A mask was used for each substrate to separate the active area for each device to either 0.25 or 1 cm^2^.

### Thin-Film Characterization

Transmission–reflection measurements were performed on a Bruker Vertex 80v Fourier transform interferometer, with a tungsten-halogen near-infrared source, a CaF_2_ beam splitter, and a silicon diode detector.

XRD patterns were measured with a Panalytical X’pert powder diffractometer with copper X-ray source (Cu K_α_ 1.54 Å set at 40 kV and 40 mA).

PL measurements were performed through photoexcitation of ITO/HTL/perovskite thin films with a 398 nm continuous wave laser (PicoHarp, LDH-D-C-405M) with a power density of 6.38 W/cm^2^ from the perovskite side. The emitted PL was coupled into a grating monochromator (Princeton Instruments, SP-2558) and measured with an ICCD camera (Princeton Instruments, PI-MAX4).

TCSPC measurements were carried out through photoexcitation of ITO/HTL/perovskite thin films with a 398 nm pulsed semiconductor diode laser (PicoHarp, LDH-D-C-405M) with a repetition rate of 10 MHz from the perovskite side. The emitted PL was coupled into a grating monochromator (Princeton Instruments, SP-2558) and collected by a photocounting detector (PDM series from MPD). Timing was controlled by a PicoHarp300 event timer.

AFM measurements were carried out using an Asylum MFP3D (Asylum Research and Oxford Instruments Co.) in AC (tapping) mode. Olympus AC240-TS-R3 silicon tips were used for topography measurements. FEI Quanta 600 FEG was used to take all SEM images. Prior to all measurements, the chamber was pumped down to high vacuum with a pressure of less than 2 × 10^–4^ mbar.

## Supporting Information Available

Further details of device fabrication and experimental procedures, including additional measurements and data of device statistics, device stability, optical characterizations, and AFM, SEM, and XRD spectra (PDF)
