# Supplementary material for: Thermally Stable Perovskite Solar Cells by All-Vacuum Deposition
Source: ACS Appl Mater Interfaces. 2022 Dec 23;15(1):772–81. doi: 10.1021/acsami.2c14658 (PMC9837819; doi:10.1021/acsami.2c14658)
Supplement: Supplementary file 1 — am2c14658_si_001.pdf [file am2c14658_si_001.pdf]

Supporting Information:
Thermally Stable Perovskite Solar Cells by All-Vacuum Deposition

Qimu Yuan[1], Kilian B. Lohmann[1], Robert D. J. Oliver[1], Alexandra J. Ramadan[1,2], Siyu Yan[1], James M. Ball[1], M. Greyson Christoforo[1], Nakita K. Noel[1], Henry J. Snaith[1], Laura M. Herz[1,3], Michael B. Johnston[1]*

[1]Department of Physics, University of Oxford, Clarendon Laboratory, Parks Road, OX1 3PU, United Kingdom
[2]Department of Physics and Astronomy, University of Sheffield, Sheffield, S3 7RH, United Kingdom
[3]Institute for Advanced Study, Technical University of Munich, Lichtenbergstrasse 2a, D-85748 Garching, Germany

E-mail: michael.johnston@physics.ox.ac.uk

Table of Contents

1. Experimental Method **2**

1.1 Device Fabrication 2

1.2 Current-Voltage Characterisation 3

1.3 Stability Testing 4

1.4 Transmission-Reflection Measurement 5

1.5 X-Ray Diffraction Measurement 5

1.6 External Quantum Efficiency Measurement 5

1.7 Steady-State Photoluminescence Measurement 5

1.8 Time-Correlated Single Photon Counting Measurement 6

1.9 Photoluminescence Quantum Yield Measurement 6

1.10 Atomic Force Microscopy Measurement 6

1.11 Scanning Electron Microscopy Measurement 6

2. Supporting Data 7

2.1 Transmission-Reflection Data of CuPc and ZnPc 7

2.2 Transmission-Reflection Data of PTAA 8

2.3 Additional Photoluminescence Results 8

2.4 Fluence-Dependent Time-Correlated Single Photon Counting and Differential Lifetime 8

2.5 Device Performance Statistics for Different Hole Transport Layers 10

2.6 *J-V* Curves of the Best Devices with Different Hole Transport Layers 10

2.7 External Quantum Efficiency Spectra of Full Devices with CuPc or ZnPc 11

2.8 Tabulated Precursor Tooling Factors on Different Substrates 11

2.9 Atomic Force Microscopy Images of Thin FAI Films Deposited on CuPc or PTAA 11

2.10 Atomic Force Microscopy Images of Thin Perovskite Films Deposited on CuPc or PTAA 12

2.11 Tabulated Summary of Root Mean Squared Roughness from Atomic Force Microscopy Measurements 13

2.12 Top-down Scanning Electron Microscopy Results 13

2.13 Cross-sectional Scanning Electron Microscopy Results 13

2.14 X-Ray Diffraction Data of $FA_{0.83}Cs_{0.17}PbI_3$ with Different PbI_2 Evaporate Rates 14

2.15 Current-Voltage Data of the Champion 1 cm^2 Device 15

2.16 Normalised Shelf-Life Stability Results of the Champion Devices 15

2.17 External Quantum Efficiency Comparison for Champion Device Stability 16

2.18 Additional Shelf-Life Stability Results from the Champion Device Batch 17
2.19 Additional Shelf-Life Stability Results from Different Batches 17
2.20 Shelf-life Stability of Devices with PTAA as Hole Transport Layer 18
2.21 Additional 85°C Oven Aging Results 19
2.22 Normalised and Averaged 85°C Oven Aging Results 19
2.23 Ambient Air Stability Results 20
2.24 X-Ray Diffraction Analysis of Device Degradation from Ambient Air Aging 20
2.25 Expected Perovskite Layer Thickness on Hole Transport Layer 21
2.26 Mobility of Hole Transport Layer from Literature Reports 21
Reference 22

1. Experimental Method

1.1 Device Fabrication

ITO substrates were gently brushed in Decon-90 detergent solution (1% volume in de-ionised water), and then sonicated for 5 minutes each in fresh Decon-90 solution, de-ionised water, acetone, and isopropyl alcohol sequentially. Before deposition, the substrates were further UV-Ozone treated for 15 minutes.

For fabricating solar-cell devices, the hole transport layers (HTLs) of Copper Phthalocyanine (CuPc) and Zinc Phthalocyanine (ZnPc), the perovskite layer, the electron transport layer C_{60}, and the buffer layer Bathocuproine (BCP) were all evaporated in the same custom-built thermal evaporator. The chamber was pumped down to a base pressure between 8 x 10^{-7} mbar and 2 x 10^{-6} mbar for all depositions. The walls of the chamber were maintained at 17 °C and the rotating substrate at 20 °C through two separate chillers. Rates were monitored through gold-plated quartz crystal microbalances (QCMs) and a customised control software. During all depositions, QCM readings at each source and at the substrate were cross-checked. Each precursor material was individually calibrated on cleaned ITO substrates (or other underlying layers, where applicable) to determine the tooling factor, and hence, the actual deposition rate.

CuPc (Sigma-Aldrich, >99.95% trace metal basis, triple-sublime grade) was evaporated at a rate of 0.08 Å/s at temperatures between 320 °C and 340 °C until a layer thickness of 7.5 nm was achieved.

ZnPc (Lumtec, >99%, sublime grade) was evaporated at a rate of 0.08 Å/s at temperatures between 300 °C and 330 °C until a layer thickness of 7.5 nm was achieved.

The Poly[bis(4-phenyl)(2,5,6-trimethylphenyl)amine (PTAA) transport layer was solution-processed in a glovebox under N_2 atmosphere. The PTAA powder (Xi'an Polymer Light Technology) was dissolved in toluene solvent at a concentration of 1.5 mg/ml, stirred overnight, and spin-coated statically with 100 μL of solution at 6000 rpm for 30 s with an acceleration of 2000 rpm, followed by 10 minutes annealing at 100 °C on a hotplate in the same N_2 atmosphere.

For the perovskite layer, $CH(NH_2)_2I$ (Dynamo, 99.999%) (FAI), PbI_2 (Alfa-Aeser, 99.998% metal base), and CsI (Alfa-Aeser, 99.998% metal base) were co-evaporated to form the $FA_{0.83}Cs_{0.17}PbI_3$ composition. To form the precise stoichiometric $FA_{0.83}Cs_{0.17}PbI_3$ composition on CuPc, FAI was evaporated at 0.2 Å/s (155 °C – 170 °C), PbI_2 was evaporated at 0.3 Å/s (270 °C – 290 °C), and CsI was evaporated at 0.04 Å/s (400 °C – 430 °C). To introduce different amounts of PbI_2 excess in the bulk perovskite, a 5%, 10%, 15%, and >20% increase of the stoichiometric PbI_2 evaporation rate was used, respectively (i.e. 0.315 Å/s, 0.33 Å/s, 0.345 Å/s, and >0.36 Å/s). For each deposition, FAI powder in the crucible was topped up to 1.1 g, and for a 520 nm perovskite layer, 0.19 – 0.21 g of FAI was typically evaporated. All as-deposited films were further annealed at 135 °C for 30 minutes on a hotplate under N_2 glovebox condition.

Fullerene C_{60} (Acros Organics, 99.9%) was deposited at 0.1 Å/s to form a 23 nm thick layer for devices with Ag contact, and 30 nm for devices with Au contact. Subsequentially, a 2 nm or 5 nm thick BCP (Sigma-Aldrich, 99.5%) layer was deposited at 0.07 Å/s for devices with Ag contact or Au contact respectively.

The Ag top contact with a thickness of 100 nm was evaporated in a separate Lesker Nano36 chamber. Using QCM readings, the initial rate was maintained at 0.2 Å/s for the first 10 nm, before ramping up to 1.5 Å/s.

The Au top contact with a thickness of 100 nm was evaporated in the same aforementioned Lesker Nano36 chamber. The initial rate was maintained at 0.1 Å/s for the first 10 nm, before ramping up to 0.7 Å/s.

1.2 Current-Voltage (*J-V*) Characterisation

Devices were measured under stimulated AM1.5G sunlight with an equivalent irradiance of 100 mW/cm^2, generated by a Wavelabs Sinus-220 solar simulator and a Keithley 2400 source meter. The solar simulator was calibrated with respect to a KG-3 filtered silicon reference photodiode (Fraunhofer) prior to the measurement. Devices were characterised in ambient air condition at room temperature with relative humidity between 25% and 40%. The open-circuit voltage (V_{oc}) was first measured for 3 s. Reverse and forward scans between -1.2 V and 0.2 V at a constant scan rate of 0.13 V/s were sequentially performed. Steady-state current and voltage were further probed for 30 s under continuous illumination, keeping the device close to its maximum power point (MPP) by actively tracking the maximum power point with a gradient descent algorithm. Finally, short-circuit current density (J_{sc}) was

measured for 3 s. A mask was used for each substrate to separate the active area for each device to either 0.25 cm^2 or 1 cm^2.

A spectral mismatch factor (M) was also estimated according to a previously reported method [1]. M was calculated to be 1.022 for devices with CuPc HTL and 1.020 for devices with PTAA and ZnPc HTL. We estimate the systematic error of this setup to be on the order of ±5% (relative). The mismatch factor has been applied to all power conversion efficiency (PCE) data points presented in this work.

1.2.1 Device Conditioning

As we note that the performance of devices tends to improve slightly with time, all device statistics shown in this work, except otherwise specified, were from a second *J-V* measurement taken between 7 and 11 days after the top metal contacts were deposited (i.e. completed fabrication). Meanwhile, PCE was calculated from the MPP of the reverse scan *J-V* curve for all data points, unless otherwise stated.

1.3 Stability Testing – Storage and Measurement Conditions

1.3.1 N_2 Atmosphere Shelf-life Stability

Unencapsulated devices were stored in a glovebox under N_2 atmosphere and under dim-light illumination. For this study, all first data points at 24 hours (h) or “day 1” equivalently denote PCE from MPP of the measured *J-V* curves tested 1 day after devices’ completed fabrication. All further PCE data points were normalised with respect to their day 1 value.

1.3.2 Environmental and Operational Stability Testing

For all devices used in the following series of stability studies, devices were not placed in the specified testing conditions until 7 days after the top metal contacts were deposited, therefore allowing a second *J-V* measurement to be taken and be consistent with the rest of the device statistics. All further PCE data were normalised with respect to this measured PCE on day 7.

1.3.2.1 N_2 Atmosphere 85 °C Oven Stability

Unencapsulated devices were placed in a sample holder and covered by a metal lid inside a home-made oven maintained at (85 ± 3)°C. The oven was kept inside the glovebox under N_2 atmosphere. Prior to every *J-V* measurement, all devices were retrieved from the oven and left inside the glovebox for additional 15 minutes to ensure they had returned to ambient temperature.

1.3.2.2 Ambient Air Stability

Unencapsulated devices were kept in dark in ambient air, at room temperature of (24 $\pm$ 3) °C, and a relative humidity between 30% and 40%.

1.4 Transmission – Reflection Measurement

Transmission – reflection measurements were performed on a Bruker Vertex 80v Fourier Transform Interferometer, with a tungsten-halogen near-infrared source, a CaF_2 beam splitter, and a silicon diode detector. A blank z-cut quartz substrate and a silver mirror were used as the transmission and the reflection reference respectively. To calculate the absorption coefficient (α), the following equation was used:

$$\alpha = -\frac{1}{l} \times \ln(\frac{T}{1-R}) \qquad (1)$$

where T is transmission, R is reflection, and l is the thickness of the deposited film, which was deduced from stylus profiler measurements.

1.5 X-Ray diffraction (XRD) Measurement

XRD patterns were measured with a Panalytical X'pert powder diffractometer with copper x-ray source (Cu-K_α 1.54 Å set at 40 kV and 40 mA). All samples measured were deposited on ITO substrates, and all spectrum were further corrected with reference to the ITO peak at 2θ = 30.4°.

1.6 External Quantum Efficiency (EQE) Measurement

EQE of fabricated devices was measured on a custom-built Fourier Transform photocurrent spectrometer with a Bruker Vertex 80v Fourier Transform Interferometer and a near-infrared source. Each device was held in place by a metal mask holder, so the exact same active area of 0.25 cm^2 (for the larger 1 cm^2 devices, only 0.25 cm^2 was sampled for the EQE measurement) as the *J-V* characterisation was illuminated. To calculate the EQE, the measured spectra was divided by the spectra of a calibrated silicon reference cell from Newport. For plotting the EQE spectrum, a smoothing function, taking the average of every nearest five data points, was also applied. To determine the J_{sc} from the spectrum EQE, the following integral was used,

$$J_{sc} = q \int_0^\infty EQE\ (\lambda)\ \varphi_{AM1.5}(\lambda)\ d\lambda \qquad (2)$$

where q is elementary charge, λ is the wavelength, and $\varphi_{AM1.5}(\lambda)$ is the AM1.5 photon flux.

1.7 Steady-State Photoluminescence (PL) Measurement

PL measurements were performed through photoexcitation of ITO/HTL/Perovskite thin films with a 398 nm continuous wave laser (PicoHarp, LDH-D-C-405M) with a power density of 6.38 W/cm^2 from the perovskite side. The emitted PL was coupled into a grating monochromator (Princeton Instruments, SP-2558) and measured with an ICCD camera (Princeton Instruments, PI-MAX4).

1.8 Time-Correlated Single Photon Counting (TCSPC) Measurement

TCSPC measurements were carried out through photoexcitation of ITO/HTL/Perovskite thin films with a 398 nm pulsed semiconductor diode laser (PicoHarp, LDH-D-C-405M) with a repetition rate of 10 MHz from the perovskite side. The emitted PL was coupled into a grating monochromator (Princeton Instruments, SP-2558) and collected by a photo-counting detector (PDM series from MPD). Timing was controlled by a PicoHarp300 event timer. Various excitation fluences were measured.

For fitting of the measured TCSPC transients, to account for the dual processes of charge transport into HTL and interfacial recombination, a double exponential in the form of $I = A_1 \exp\left(\frac{-t}{\tau_1}\right) + A_2 \exp\left(\frac{-t}{\tau_2}\right)$ was used.

1.9 Photoluminescence Quantum Yield (PLQY) Measurement

For PLQY measurements, ITO/HTL/Perovskite samples were placed in an integrating sphere and photo-excited by a 532 nm laser. The illumination intensity was 24 mW/cm^2 and equivalent to the half sun intensity. The signal was collected by a QEPro spectrometer.

1.10 Atomic Force Microscopy (AFM) Measurement

AFM measurements were carried out using an Asylum MFP3D (Asylum Research and Oxford Instruments Co.) in AC (tapping) mode. Olympus AC240-TS-R3 silicon tips were used for topography measurements.

1.11 Scanning Electron Microscopy (SEM) Measurement

FEI Quanta 600 FEG was used to take all SEM images. Prior to all measurements, the chamber was pumped down to high vacuum with a pressure less than 2x10^{-4} mbar. For top-down SEM images, an acceleration voltage of 2 keV was chosen and current was defined by a spot size of 2.5.

2. Supporting Data

2.1 Transmission – Reflection Data of CuPc and ZnPc

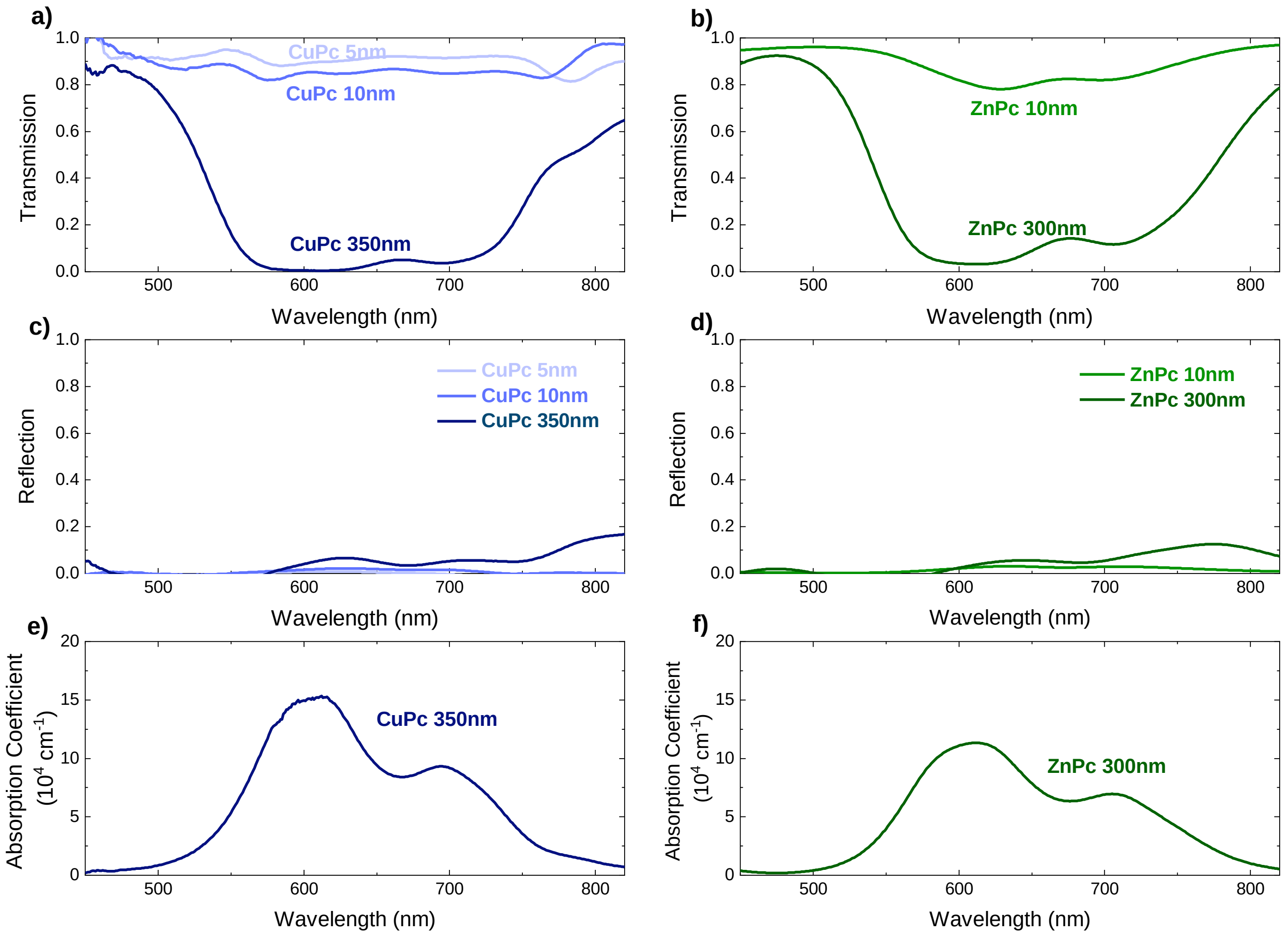

Figure S1: a) Transmission of CuPc thin films of various thickness deposited on z-cut quartz; **b)** Transmission of ZnPc thin films of various thickness deposited on z-cut quartz; **c)** Reflection of CuPc thin films of various thickness deposited on z-cut quartz; **d)** Reflection of ZnPc thin films of various thickness deposited on z-cut quartz; **e)** Absorption coefficient of the 350 nm thick CuPc film deposited on z-cut quartz; **f)** Absorption coefficient of the 300 nm thick ZnPc film deposited on z-cut quartz.

2.2 Transmission – Reflection Data of PTAA

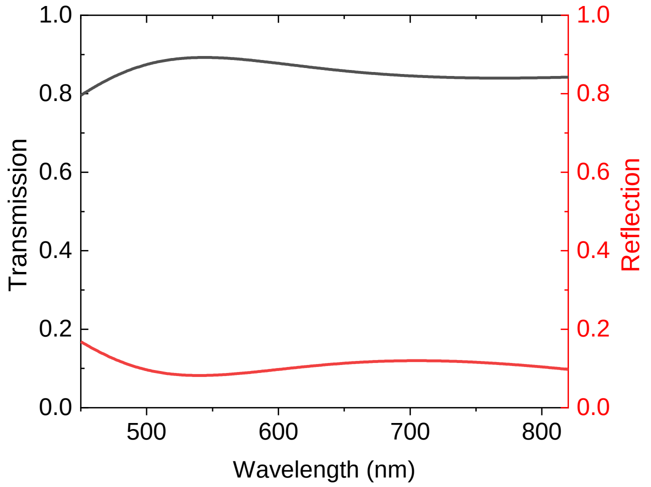

Figure S2: Transmission (black) and reflection (red) spectra of the spin-coated PTAA thin film, deposited on ITO substrate and used as hole transport layer in this study.

2.3 Additional PL Results

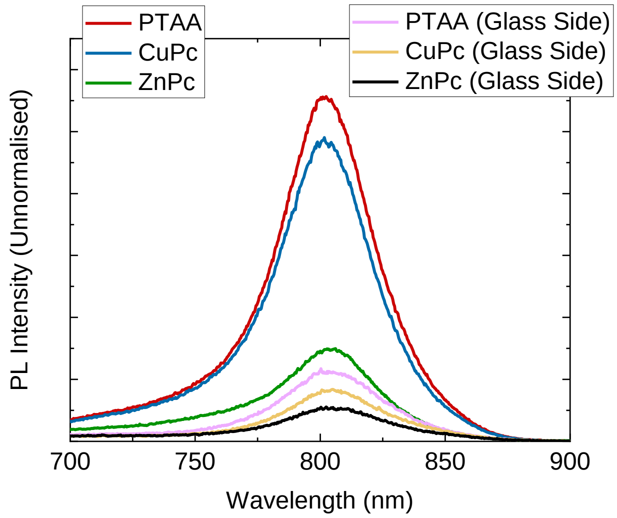

Figure S3: Unnormalised photoluminescence spectra of the ITO/HTL/$FA_{0.83}Cs_{0.17}PbI_3$ half-stacks for three HTLs of PTAA, CuPc, or ZnPc. Laser excitation and photoluminescence collection were performed from both the "front" (i.e. direct excitation of the perovskite top layer) and the "glass side" (i.e. laser passing through the glass substrate, ITO and HTL first before exciting the perovskite). A 398 nm wavelength laser was used to excite the perovskite in all cases.

2.4 Fluence-dependent TCSPC and Differential Lifetime

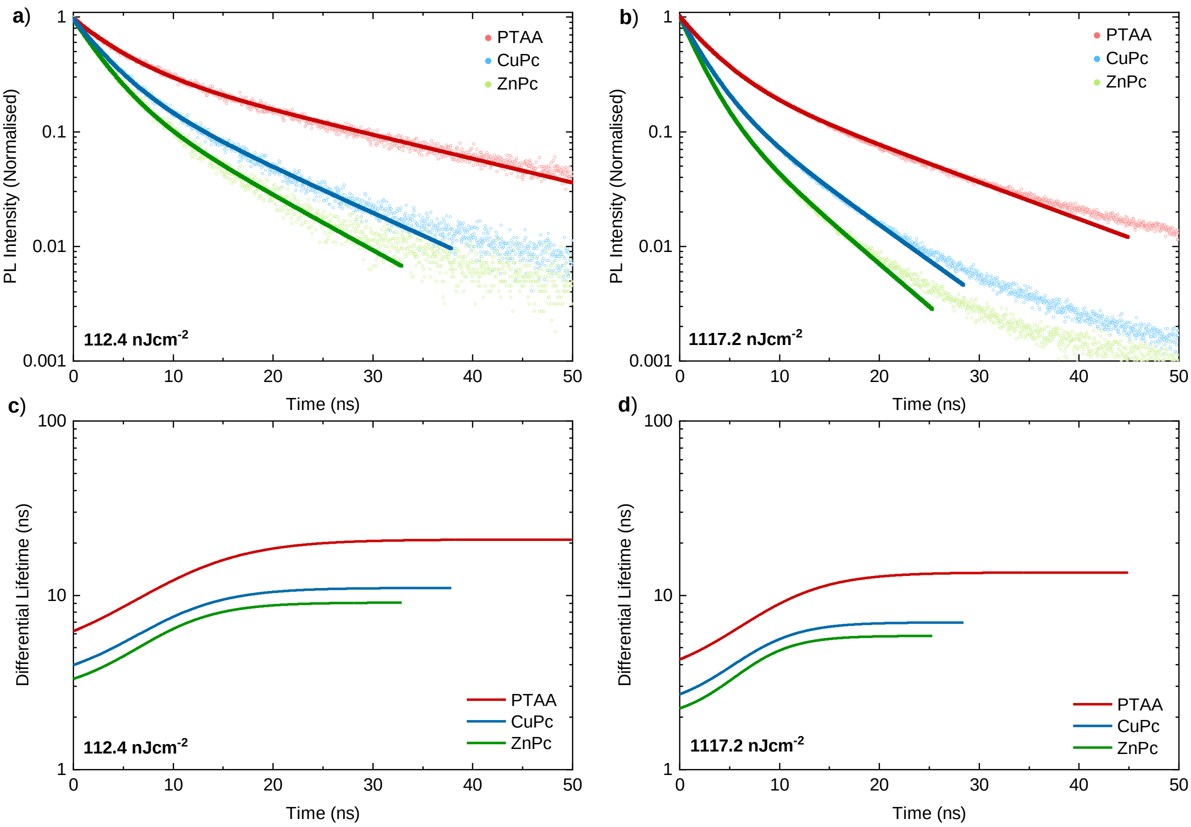

Figure S4: Time resolved PL transients of $FA_{0.83}Cs_{0.17}PbI_3$ part-solar-cell devices with three different hole transport layers (HTLs) of PTAA (red), CuPc (blue), and ZnPc (green). The layer architecture was ITO/HTL/$FA_{0.83}Cs_{0.17}PbI_3$ (520 nm). The samples were excited by a 398 nm semiconductor pulsed diode laser with a repetition frequency of 10 MHz. **a)** Illustrates PL transient from an excitation fluences of 112.4 nJ/cm^2 and **b)** from an excitation fluences of 1117.2 nJ/cm^2. The resulting PL was collected and passed through a spectrometer to select the PL peak wavelength centred around 800 nm before detection on a Si avalanche photodiode. Time resolution was obtained using the technique of TCSPC. **c, d)** Differential lifetime for each excitation fluence calculated using equation 2 in the main text and an analysis method outlined in section 8 of the experimental method.

2.5 Device Performance Statistics for Different HTLs

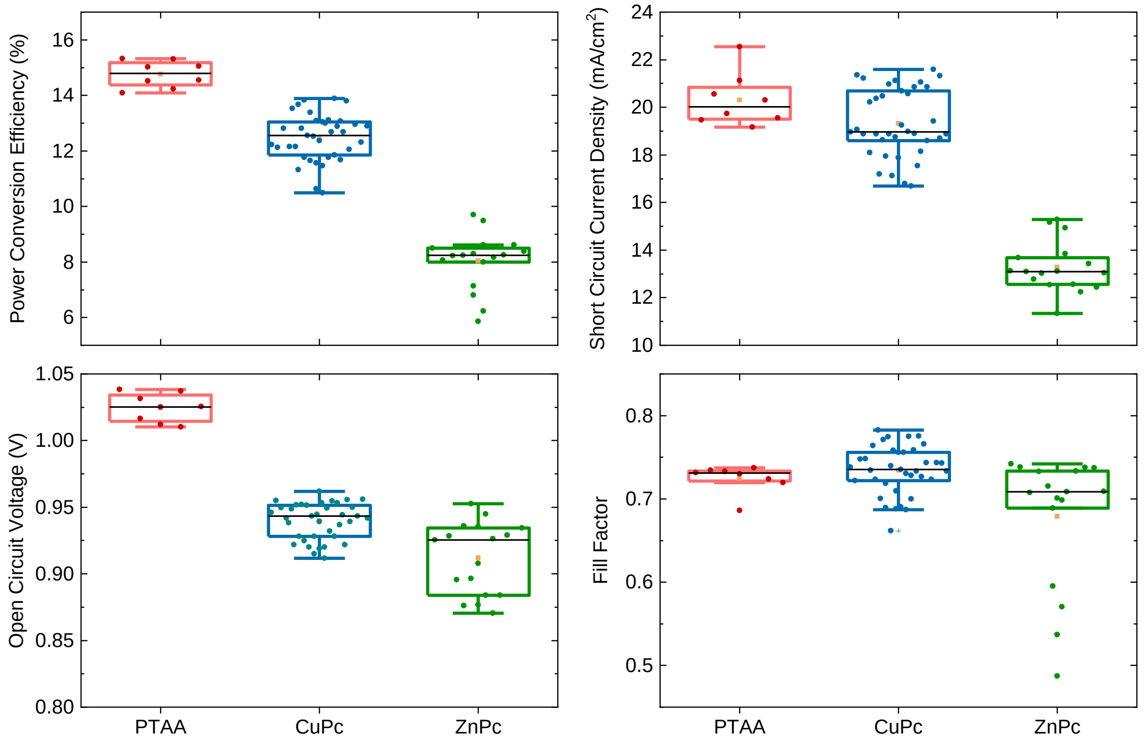

Figure S5: Device performance statistics of the ITO/HTL/$FA_{0.83}Cs_{0.17}PbI_3$ (520 nm)/C_{60} (23 nm)/BCP (2 nm)/Ag (100 nm) solar cells, where HTL is PTAA, CuPc (7.5 nm), or ZnPc (7.5 nm). Mean and median are denoted by an orange square and a black line respectively.

2.6 *J-V* Curves of both Forward and Reverse Scans of the Best Devices with Different HTL

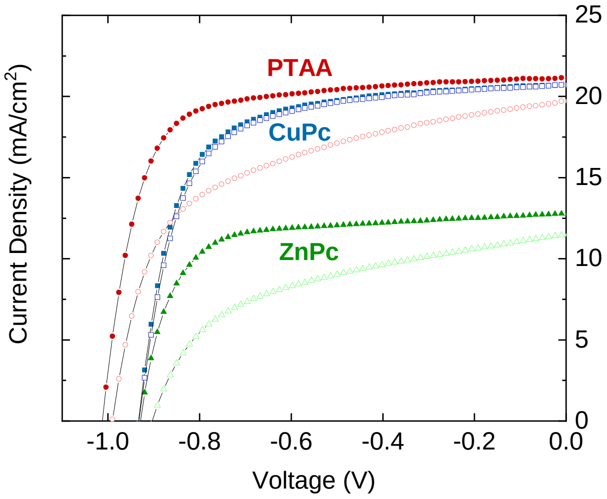

Figure S6: J-V curves of the best ITO/HTL/$FA_{0.83}Cs_{0.17}PbI_3$/C_{60}/BCP/Ag devices, where HTL is PTAA (red circle), CuPc (blue square), or ZnPc (green triangle). Forward scans are plotted with hollow symbols and reverse scans are denoted by solid symbols. A scan rate of 0.13 V/s was used for all scans.

2.7 EQE Spectra of Full Devices with CuPc or ZnPc HTL

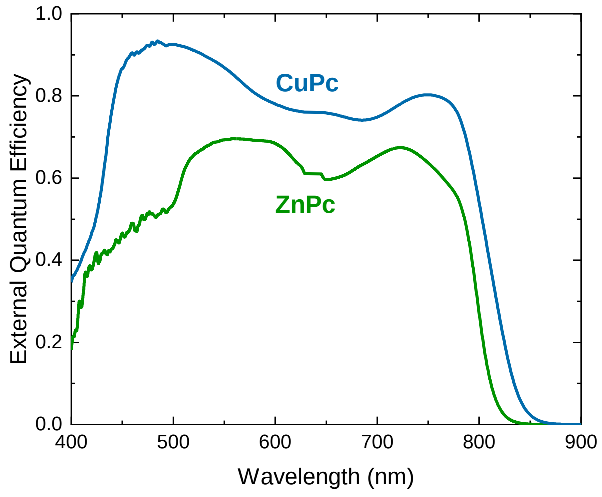

Figure S7: EQE spectra of the champion all-evaporated ITO/HTL (7.5 nm)/$FA_{0.83}Cs_{0.17}PbI_3$ (520 nm)/C_{60} (23 nm)/BCP (2 nm)/Ag (100 nm) devices, where HTL is CuPc or ZnPc.

2.8 Tabulated Precursor Tooling Factors on Different Substrates

Two additional substrates of clean ITO and N,N′-di(1-naphthyl)-N,N′-diphenyl-(1,1′-biphenyl)-4,4′-diamine (NPB) were also probed. NPB (Lumtec, >99.5%, sublimed, HPLC) was evaporated at a rate of 0.1 Å/s at temperature between 165 °C and 185 °C until a layer thickness of 10 nm was achieved.

To determine the actual thickness of each deposited film, at minimum 20 stylus profiler measurements were taken across different sections of the film, and an average was calculated. We calculated the tooling factor according to the following equation:

$$Tooling\ Factor = \frac{Thickness_{\mathrm{Measured}}}{Thickness_{\mathrm{Substrate\ QCM\ Reading}}} \tag{3}$$

Precursor/ Substrates	FAI	PbI_2	CsI
PTAA	0.31 (±0.02)	0.47 (±0.03)	0.48 (±0.04)
CuPc	0.45 (±0.04)	0.48 (±0.03)	0.50 (±0.04)
ZnPc	0.49 (±0.04)	0.48 (±0.03)	0.51 (±0.04)
ITO	0.35 (±0.03)	0.46 (±0.03)	0.48 (±0.04)
NPB	0.34 (±0.03)	0.45 (±0.02)	-

Table S1: Tooling factor results for deposited precursors of FAI, PbI_2, and CsI on substrates of spin-coated PTAA, cleaned ITO, and evaporated CuPc, ZnPc, and NPB.

2.9 Additional AFM Images of Thin FAI Films Deposited on CuPc or PTAA

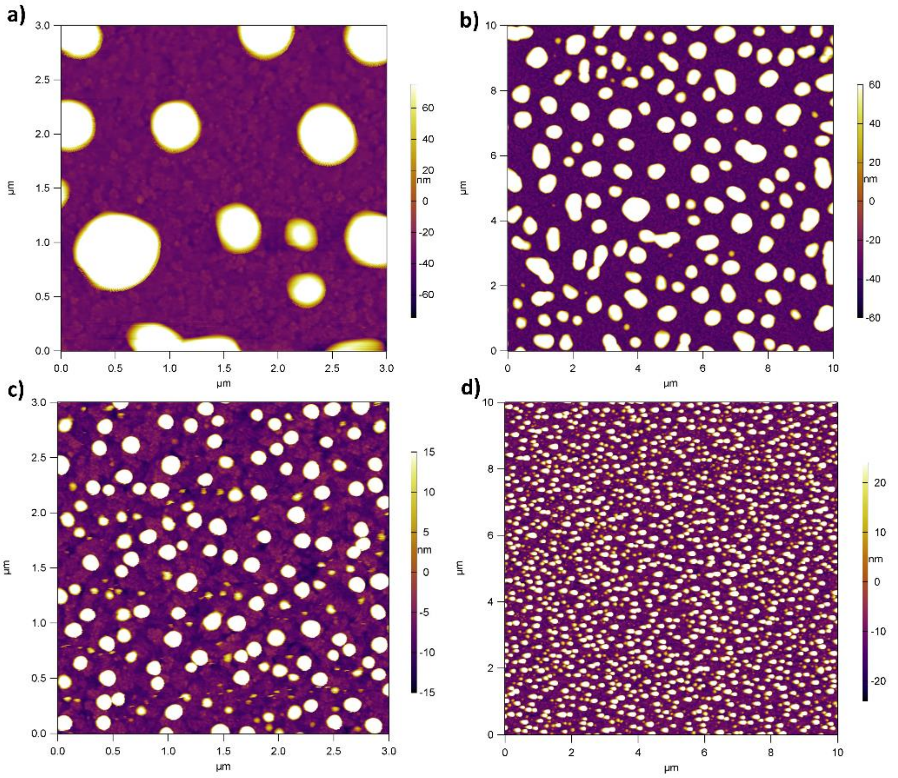

Figure S8: Additional AFM images illustrating the topography of **a)** 5 nm of FAI on CuPc; **b)** 5 nm of FAI on PTAA; **c)** 25 nm of FAI on CuPc; **d)** 25 nm of FAI on PTAA.

2.10 AFM Images of Thin Perovskite Films Deposited on CuPc or PTAA

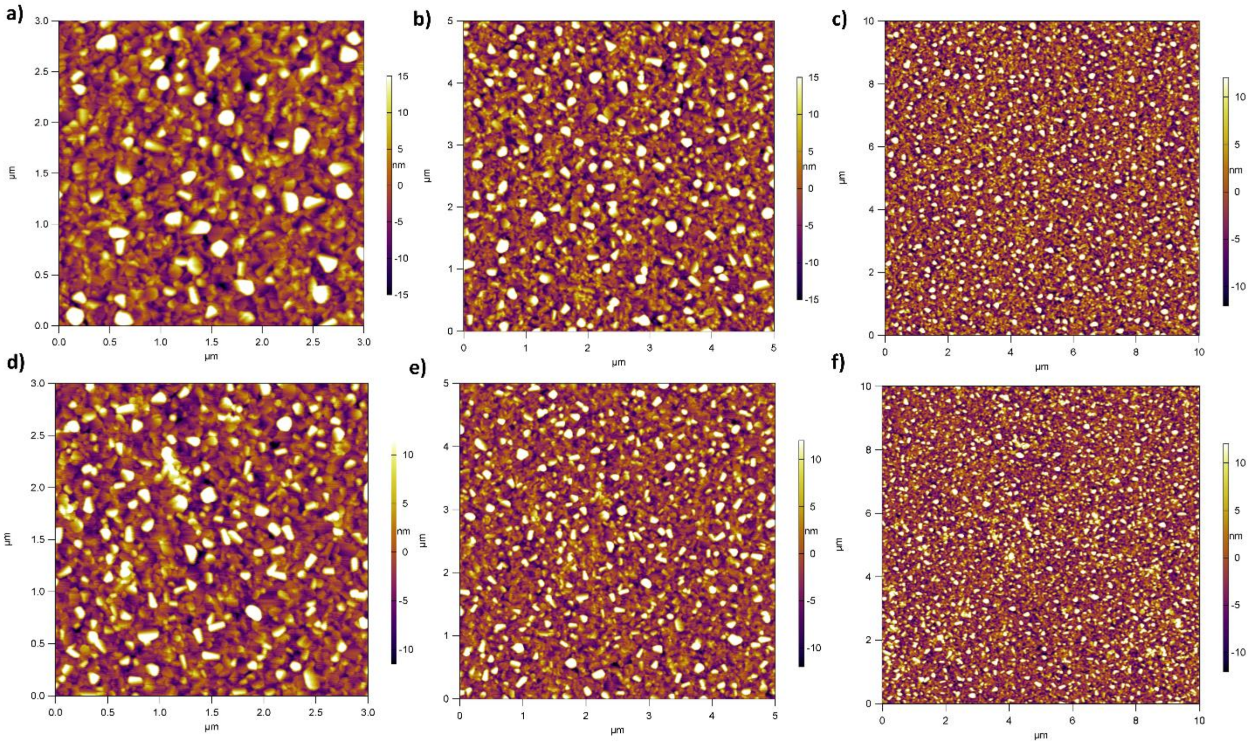

Figure S9: AFM images illustrating the topography of 25 nm $FA_{0.83}Cs_{0.17}PbI_3$ perovskite deposited on **a)**, **b)**, and **c)** evaporated CuPc, and **d)**, **e)**, and **f)** spin-coated PTAA.

2.11 Tabulated Summary of Root Mean Squared Roughness (R_q) from AFM Measurements

Sample	R_q (3 µm image) (nm)	R_q (5 µm image) (nm)	R_q (10 µm image) (nm)	Mean R_q (nm)	St. deviation error (nm)
5 nm FAI on CuPc	13.1	17.8	18.4	16.4	2.4
5 nm FAI on PTAA	2.1	2.1	2.0	2.1	0.1
25 nm FAI on CuPc	65.0	65.7	58.1	61.3	2.8
25 nm FAI on PTAA	18.1	18.2	19.0	18.4	0.4
25 nm $FACsPbI_3$ on CuPc	7.4	7.6	7.5	7.5	0.1
25 nm $FACsPbI_3$ on PTAA	5.8	5.9	6.1	5.9	0.1

Table S2: A summary of R_q values of all measured AFM images in Figure 2c, 2d, 2e and 2f, Figure S8, and Figure S9.

2.12 Top-down SEM Results

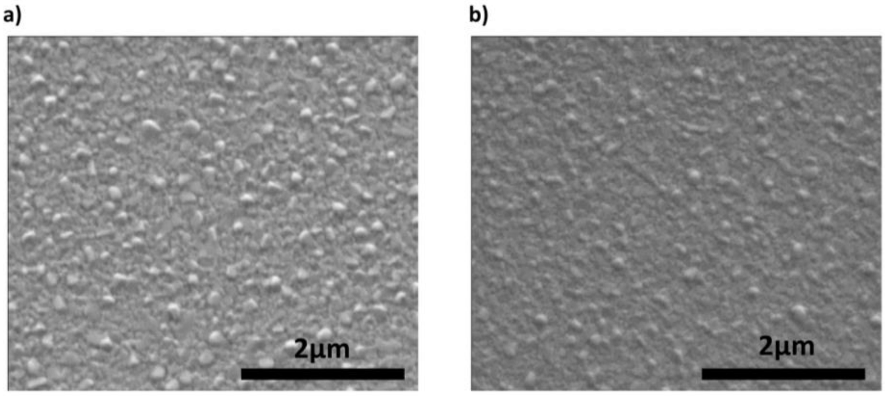

Figure S10: Top-down SEM images of co-deposited 25 nm thick $FA_{0.83}Cs_{0.17}PbI_3$ perovskite on **a)** evaporated CuPc and **b)** spin-coated PTAA. The acceleration voltage used was 2 keV.

2.13 Cross-sectional SEM Results

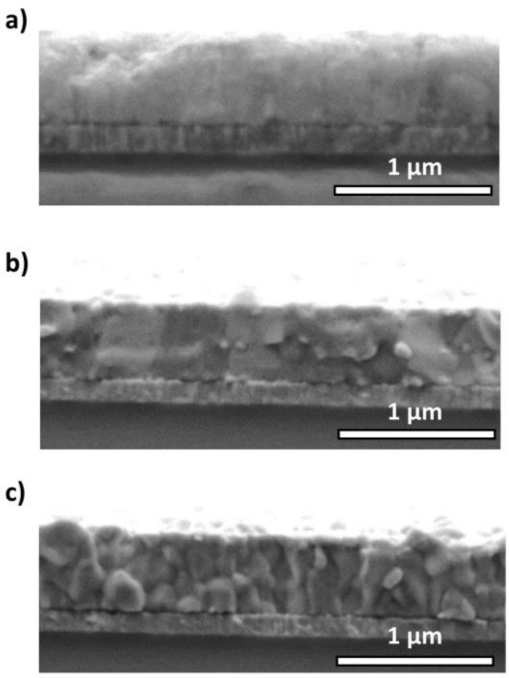

Figure S11: Cross-section SEM images of the ITO/HTL/$FA_{0.83}Cs_{0.17}PbI_3$ half-stack where HTL is **a)** spin-coated PTAA, **b)** evaporated CuPc, and **c)** evaporated ZnPc. The acceleration voltage used was 2 keV.

2.14 XRD Data of $FA_{0.83}Cs_{0.17}PbI_3$ with Different PbI_2 Evaporate Rates

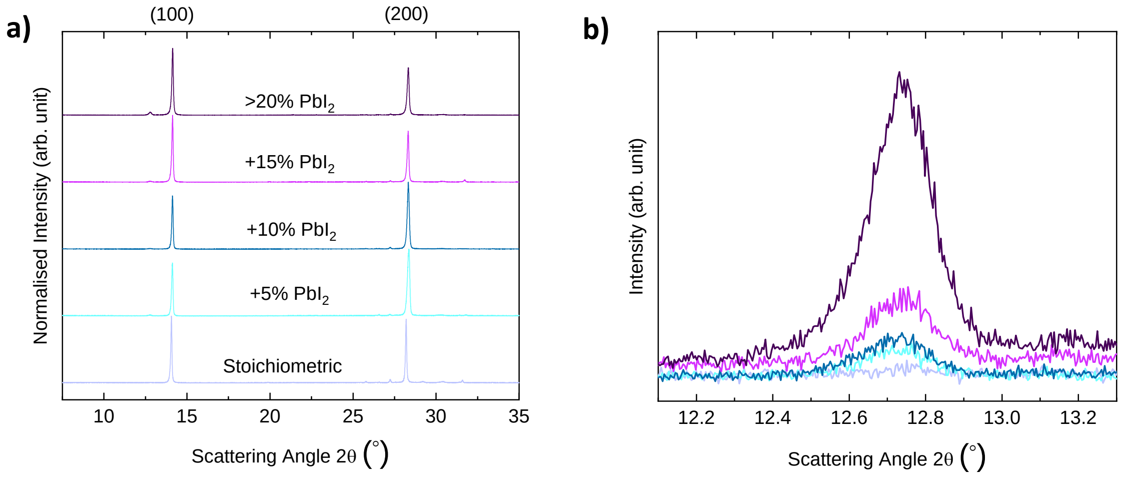

Figure S12: X-ray diffraction patterns from a Cu-Kα 1.54 Å source of the ITO/CuPc (7.5nm)/$FA_{0.83}Cs_{0.17}PbI_3$ (520 nm) half-stacks for different PbI_2 evaporation rate relative to the stoichiometric case. **a)** Illustrates the pattern of diffraction angles from 2θ of 7.5° to 35° with intensity normalised; and **b)** shows zoomed in and unnormalised PbI_2 diffraction peaks.

PbI_2 Evaporate Rates	Intensity of PbI_2 peak to ITO reference peak
Stoichiometric	0.87
5% PbI_2 rate	1.08
10% PbI_2 rate	1.44
15% PbI_2 rate	1.79
>20% PbI_2 rate	3.68

Table S3: Intensity ratio of the PbI_2 X-ray diffraction peak to the ITO reference peak for each X-ray diffraction pattern of different PbI_2 evaporation rates in Figure S12. As there is no observable PbI_2 peak for the stoichiometric case, the recorded intensity value at 2θ of 12.7° was used for calculation.

2.15 *J-V* Data of the Champion 1 cm^2 Device

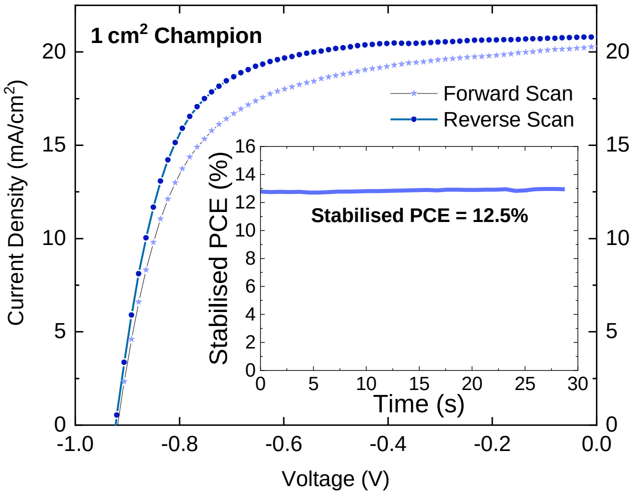

Figure S13: *J-V* plots of the champion 1 cm^2 active area ITO/CuPc (7.5nm)/$FA_{0.83}Cs_{0.17}PbI_3$ (520 nm)/C_{60} (23 nm)/BCP (2 nm)/Ag (100 nm) device with the perovskite optimised with 10% extra PbI_2 deposition rate. The maximum power point from the reverse scan gave a PCE of 12.9%, J_{sc} of 20.8 mA/cm^2, V_{oc} of 0.92 V, and fill factor of 0.69. The inset shows the stabilised power output under continuous illumination and tracked over 30 s.

2.16 Normalised Shelf-life Stability Results of the Champion Devices

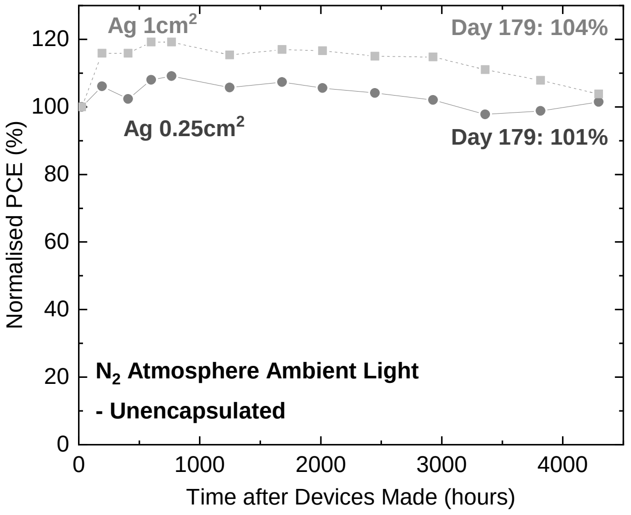

Figure S14: Normalised N_2 atmosphere shelf-life stability measurements from the champion 0.25 cm^2 and 1 cm^2 active area ITO/CuPc/$FA_{0.83}Cs_{0.17}PbI_3/C_{60}$/BCP/Ag devices. For the first measurement at day 1 (24 hours), the 0.25 cm^2- and 1 cm^2-device recorded a PCE of 13.1% and 11.2% respectively. Based on our device conditioning, the second measurement at day 8 registered a PCE of 13.9% and 12.9% respectively, and are reflected in device statistics (Figure 3a, 3b, and Figure S13) in this work.

2.17 EQE Comparison for Champion Device Stability

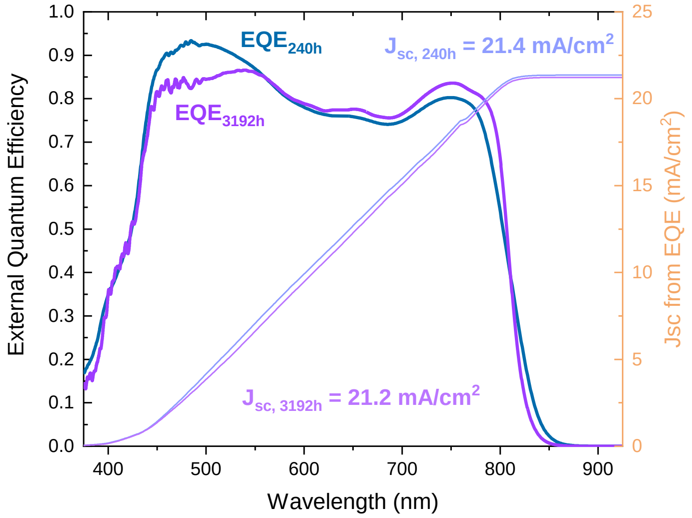

Figure S15: EQE spectra and Integrated J_{sc} from EQE of the 0.25 cm^2 ITO/CuPc/$FA_{0.83}Cs_{0.17}PbI_3/C_{60}$/BCP/Ag champion device measured at 240 hours (10 days) and 3192 hours (133 days) after its completed fabrication. Devices were stored in N_2 atmosphere as specified in section 3.1 of the experimental method.

2.18 Additional Shelf-life Stability Results from the Champion Device Batch

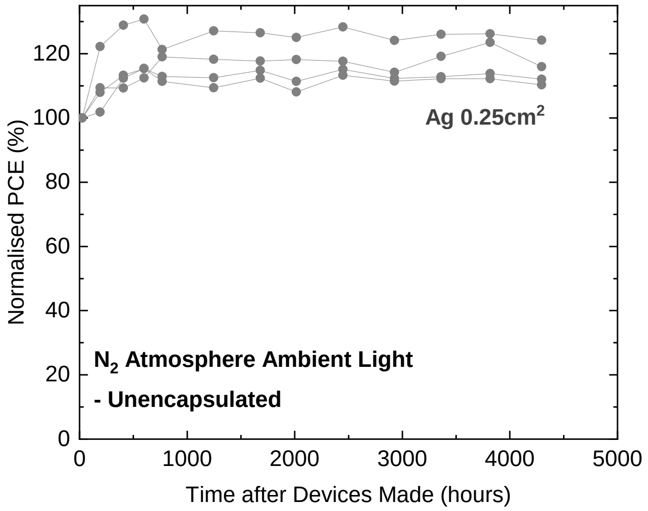

Figure S16: Additional N_2 atmosphere shelf-life stability measurements from ITO/CuPc/$FA_{0.83}Cs_{0.17}PbI_3$/C_{60}/BCP/Ag devices fabricated in the same batch as of the champion devices reported in Figure 3b, Figure S13, and Figure S14. Devices were measured to have a day 1 PCE of 12.3% (0.25 cm^2), 11.8% (0.25 cm^2), 10.9% (0.25 cm^2), and 10.2% (0.25 cm^2) from MPP.

2.19 Additional Shelf-life Stability Results from Different Batches

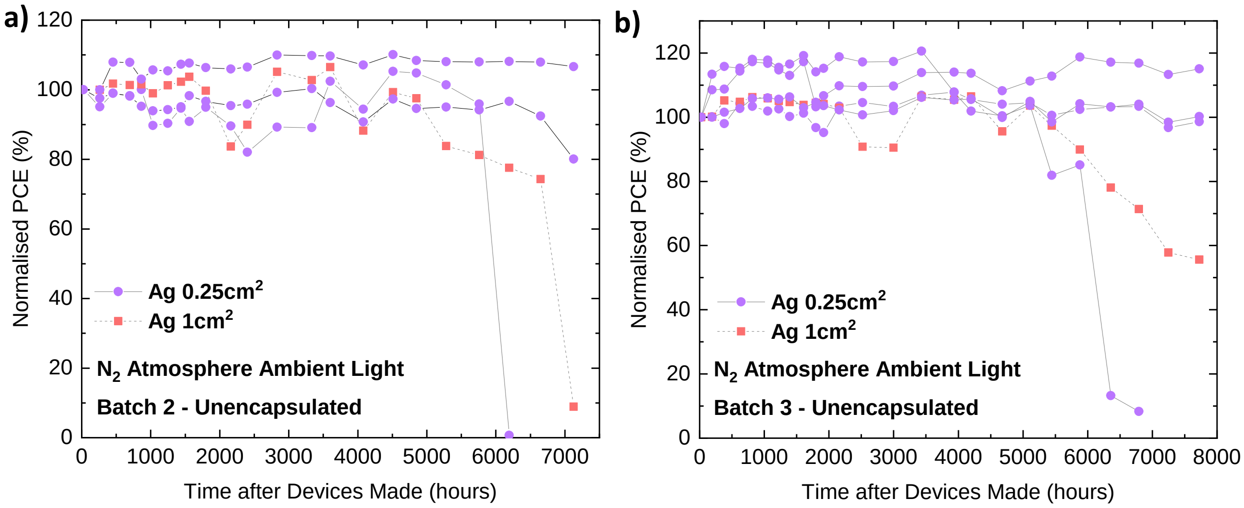

Figure S17: Additional N_2 atmosphere shelf-life stability measurements from ITO/CuPc/$FA_{0.83}Cs_{0.17}PbI_3$/C_{60}/BCP/Ag devices of various active area fabricated in separate batches. Devices were measured to have a day 1 PCE of **a)** 12.7% (0.25 cm^2), 12.4% (0.25 cm^2), 11.8%* (0.25 cm^2), and 11.9%* (1 cm^2); **b)** 10.8% (0.25 cm^2), 12.8% (0.25 cm^2), 10.8% (0.25 cm^2), 12.1%* (0.25 cm^2), and 11.7% (1 cm^2). Three devices with * registered an electrical short-circuit on the first measurement, but resumed normal operation from the second measurements at **a)** 264 hours and **b)** 192 hours, and further data points were normalised with respective to these values.

2.20 Shelf-life Stability of Devices with PTAA as HTL

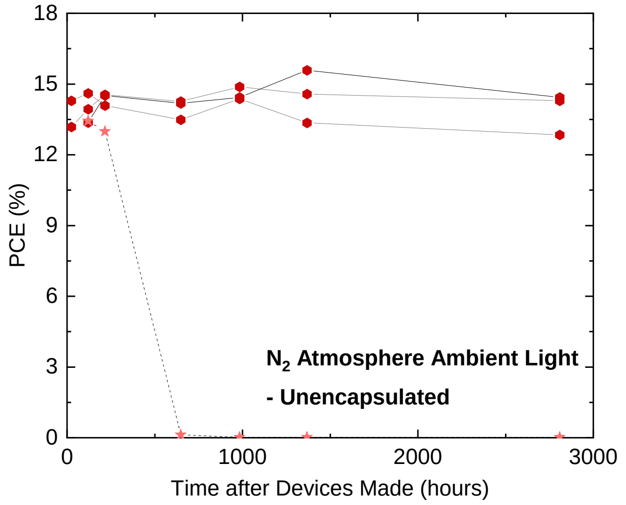

Figure S18: N_2 atmosphere shelf-life stability measurements of three 0.25 cm^2-area (red hexagon) and one 1 cm^2-area (pink star) ITO/PTAA/$FA_{0.83}Cs_{0.17}PbI_3$/C_{60}/BCP/Ag device. One 0.25 cm^2- and one 1 cm^2-device recorded an electrical short-circuit on first measurement but resumed to normal operation after.

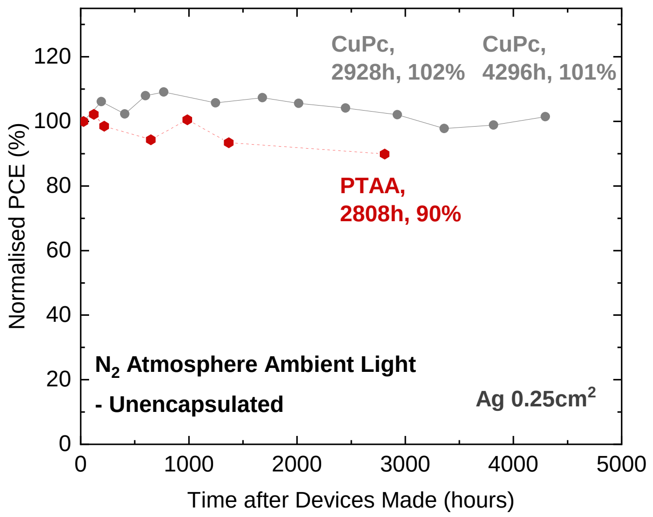

Figure S19: Normalised comparison of the champion CuPc device (also shown in Figure 4a and Figure S14) and the best PTAA device (also shown in Figure S18) used for stability studies. 0.25 cm^2-area ITO/HTL/$FA_{0.83}Cs_{0.17}PbI_3$/C_{60}/BCP/Ag devices were both stored in N_2 atmosphere in dim-light. The CuPc device (grey circle) recorded a PCE of 13.1% from first measurement, one day after its fabrication, while the PTAA device (red hexagon) registered a PCE of 14.6%.

2.21 Additional 85°C Oven Aging Results

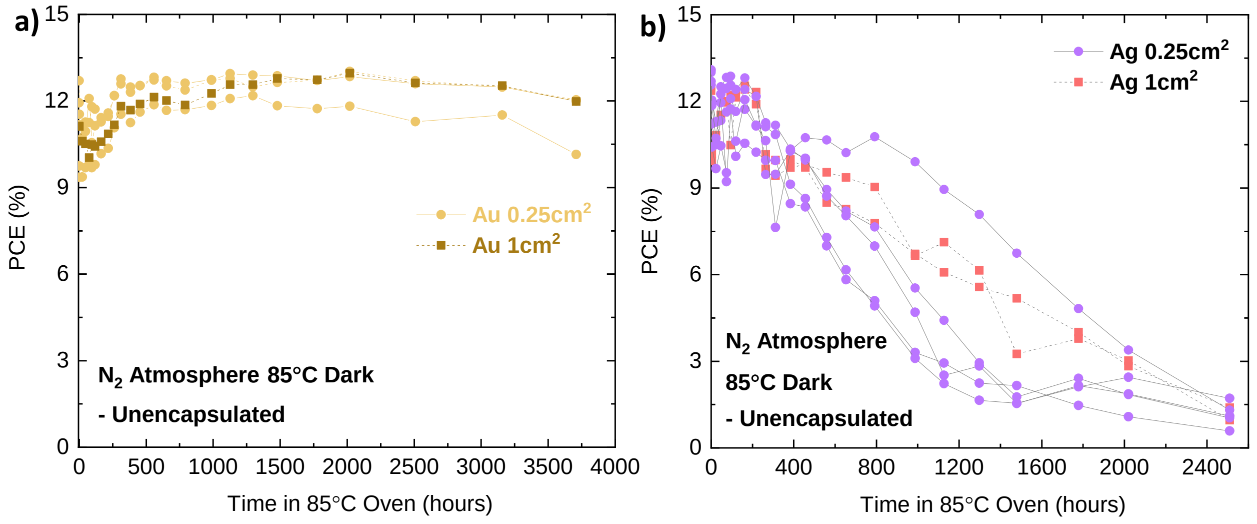

Figure S20: Additional N_2 atmosphere 85 °C oven aging results for each unencapsulated ITO/CuPc/$FA_{0.83}Cs_{0.17}PbI_3/C_{60}$/BCP devices with **a)** Au top contacts **b)** Ag top contacts reported in Figure 4b and Figure S21. Initially, devices were measured to have a PCE of **a)** 12.7% (0.25 cm^2), 11.9% (0.25 cm^2), 9.4% (0.25 cm^2), and 11.2% (1 cm^2); **b)** 13.0% (0.25 cm^2), 13.0% (0.25 cm^2), 12.5% (0.25 cm^2), 13.1% (0.25 cm^2), 12.7% (0.25 cm^2), 12.3% (1 cm^2), and 12.5% (1 cm^2). For devices with Au top contacts in **a)**, after 3710 hours, respective PCE of 12.0% (0.25 cm^2), 12.0% (0.25 cm^2), 10.2% (0.25 cm^2), and 12.0% (1 cm^2) were measured. For devices with Ag top contacts in **b)**, after 2510 hours, respective PCE of 1.3% (0.25 cm^2), 1.1% (0.25 cm^2), 0.6% (0.25 cm^2), 1.1% (0.25 cm^2), 1.8% (0.25 cm^2), 1.0% (1 cm^2), and 1.4% (1 cm^2) were measured.

2.22 Normalised and Averaged 85°C Oven Aging Results

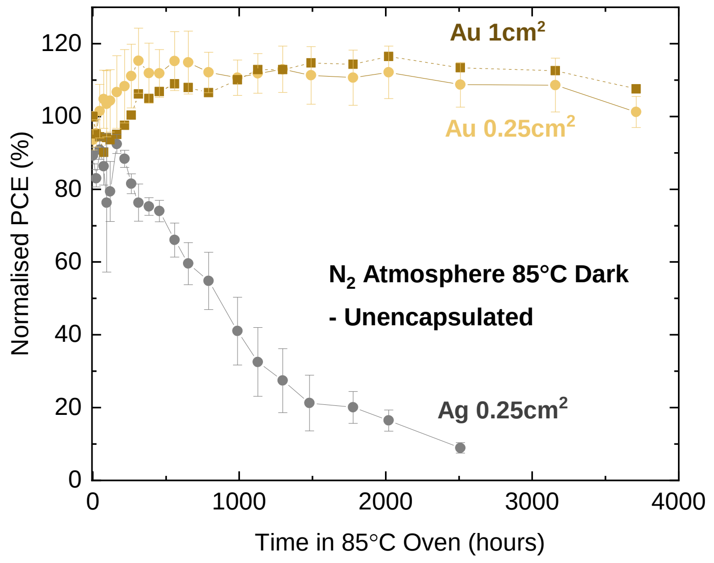

Figure S21: Normalised presentation of Figure 4b reflecting the thermal stability of unencapsulated 0.25 cm^2 and 1 cm^2 active area ITO/CuPc/$FA_{0.83}Cs_{0.17}PbI_3$/C_{60}/BCP solar-cell devices with Ag or Au metal contacts stored in a 85°C oven in N_2 atmosphere.

2.23 Ambient Air Stability Results

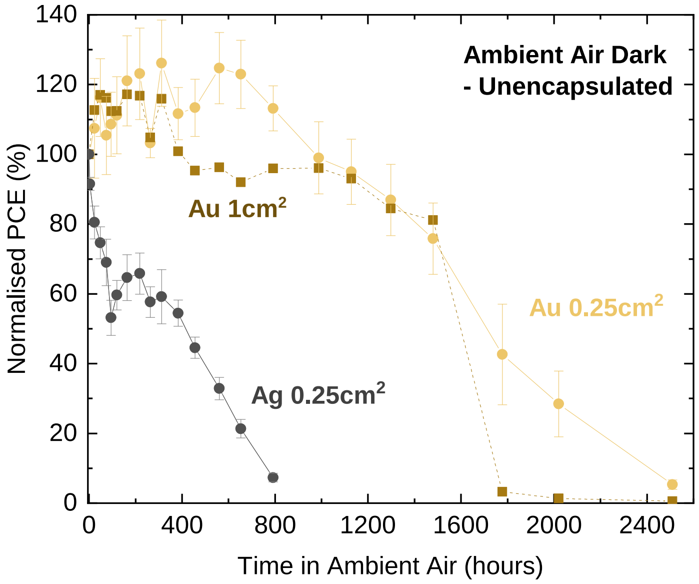

Figure S22: Ambient air stability results for unencapsulated ITO/CuPc/$FA_{0.83}Cs_{0.17}PbI_3$/ C_{60}/BCP devices with Ag or Au top contacts. For devices with an active area of 0.25 cm^2, the normalised average of 4 Ag devices and 6 Au devices (3 Au devices from 2180 hours) are illustrated. Prior to the ambient air stability testing, PCE of Ag devices were 13.7%, 13.8%, 13.5%, and 12.9%, while PCE of 0.25 cm^2 Au devices were 13.5%, 12.7%, 12.4%, 12.1%, 8.4%, and 6.8%. One normalised Au devices with 1 cm^2 active area is also plotted, which had an initial PCE of 12.3%.

2.24 XRD Analysis of Device Degradation from Ambient Air Aging

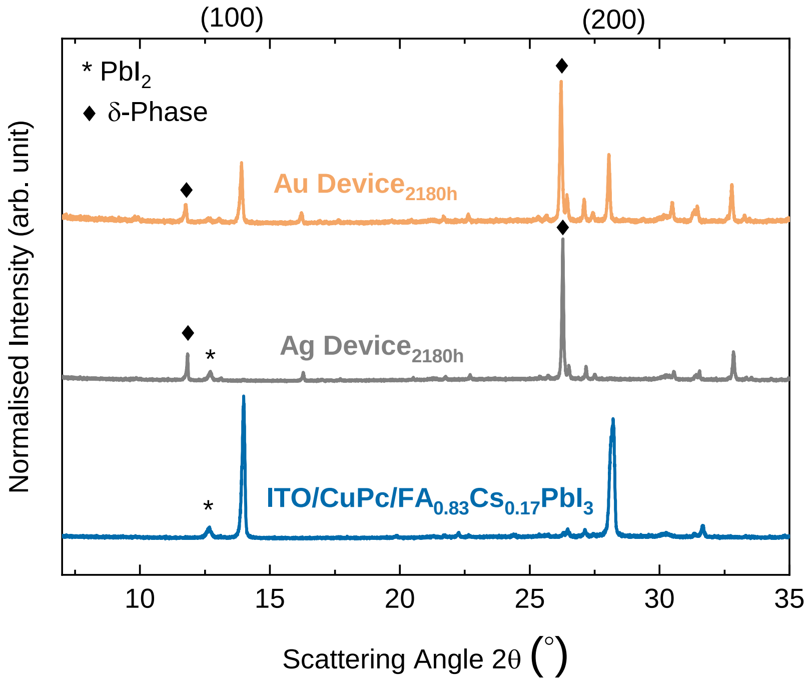

Figure S23: XRD pattern illustrating full ITO/CuPc/$FA_{0.83}Cs_{0.17}PbI_3$/C_{60}/BCP devices with Ag or Au metal contacts aged in ambient air condition for 2180 hours, in conjunction with an ITO/CuPc/$FA_{0.83}Cs_{0.17}PbI_3$ half-stack sample without any aging. For all three samples, the perovskite layer was deposited in the same batch. The X-ray source used was Cu-K_{α} 1.54 Å. For the Ag device with 2180 hours of ambient air aging, perovskite has predominantly degraded into the yellow delta-phase. For the Au device with 2180 hours of ambient air aging, both delta and tetragonal perovskite phases are observed.

2.25 Expected Perovskite Layer Thickness on Hole Transport Layer

Hole Transport Layer	Perovskite Layer Thickness (nm)
CuPc	520 ± 15
PTAA	485 ± 13
ZnPc	532 ± 15

Table S4: Expected perovskite layer thickness for co-evaporated $FA_{0.83}Cs_{0.17}PbI_3$ on different hole transport layers with respect to a 520 nm thick layer on CuPc for the same deposition time. Note that this table only provides an illustration of the impact of different precursor tooling factors on different HTLs. All perovskite layers presented in this paper, unless otherwise stated, were deposited to approximately 520 nm thick on all HTLs.

2.26 Mobility of Hole Transport Layer from Literature Reports

**HTL**	**Mobility (cm^2 V^{-1} s^{-1})**	**Reference**
**PTAA (Solution-processed)**	4×10^{-3}	Zhang *et al.* 2009 [2]
**CuPc (Thermal-evaporated)**	Order of 10^{-3}	Gao *et al.* 2007 [3]
**CuPc (Thermal-evaporated)**	5.8×10^{-3}	Kraus *et al.* 2010 [4]

**CuPc (Solution-processed)**	2.3×10^{-4}	Wang *et al.* 2017 [5]
**ZnPc (Thermal-evaporated)**	1.9×10^{-3}	Schünemann *et al.* 2012 [6]

Table S5: A short summary of selected carrier mobility values for undoped hole transport layers of PTAA, CuPc, and ZnPc from literature reports.

Reference

[1]: Snaith, H. J. How Should You Measure Your Excitonic Solar Cells?. *Energy & Environmental Science* **2012**, *5* (4), 6513-6520.

[2]: Zhang, W.; Smith, J.; Hamilton, R.; Heeney, M.; Kirkpatrick, J.; Song, K.; Watkins, S. E.; Anthopoulos, T.; McCulloch, I. Systematic Improvement in Charge Carrier Mobility of Air Stable Triarylamine Copolymers. *Journal of the American Chemical Society* **2009**, *131* (31), 10814–10815.

[3]: Gao, J.; Xu, J. B.; Zhu, M.; Ke, N.; Ma, D. Thickness Dependence of Mobility in CuPc Thin Film on Amorphous SiO_2 Substrate. *Journal of Physics D: Applied Physics* **2007**, *40* (18), 5666–5669.

[4]: Kraus, M.; Richler, S.; Opitz, A.; Brütting, W.; Haas, S.; Hasegawa, T.; Hinderhofer, A.; Schreiber, F. High-Mobility Copper-Phthalocyanine Field-Effect Transistors with Tetratetracontane Passivation Layer and Organic Metal Contacts. *Journal of Applied Physics* **2010**, *107* (9), 094503.

[5]: Wang, J.-M.; Wang, Z.-K.; Li, M.; Zhang, C.-C.; Jiang, L.-L.; Hu, K.-H.; Ye, Q.-Q.; Liao, L.-S. Doped Copper Phthalocyanine via an Aqueous Solution Process for Normal and Inverted Perovskite Solar Cells. *Advanced Energy Materials* **2018**, *8* (2), 1701688.

[6]: Schünemann, C.; Wynands, D.; Wilde, L.; Hein, M. P.; Pfützner, S.; Elschner, C.; Eichhorn, K.-J.; Leo, K.; Riede, M. Phase Separation Analysis of Bulk Heterojunctions in Small-Molecule Organic Solar Cells Using Zinc-Phthalocyanine and C_{60}. *Physical Review B* **2012**, *85* (24), 245314.
